# Health and Environmental Justice Implications of Retiring Two Coal‐Fired Power Plants in the Southern Front Range Region of Colorado

**DOI:** 10.1029/2019GH000206

**Published:** 2019-09-26

**Authors:** Sheena E. Martenies, Ali Akherati, Shantanu Jathar, Sheryl Magzamen

**Affiliations:** ^1^ Department of Environmental and Radiological Health Sciences Colorado State University Fort Collins CO USA; ^2^ Department of Mechanical Engineering Colorado State University Fort Collins CO USA; ^3^ Department of Epidemiology Colorado School of Public Health Fort Collins CO USA

**Keywords:** health impact assessment, environmental justice, inequality metrics, ambient air pollution, economic assessment, community multiscale air quality model

## Abstract

Despite improvements in air quality over the past 50 years, ambient air pollution remains an important public health issue in the United States. In particular, emissions from coal‐fired power plants still have a substantial impact on both nearby and regional populations. Of particular concern is the potential for this impact to fall disproportionately on low‐income communities and communities of color. We conducted a quantitative health impact assessment to estimate the health benefits of the proposed decommissioning of two coal‐fired electricity generating stations in the Southern Front Range region of Colorado. We estimated changes in exposures to fine particulate matter and ozone using the Community Multiscale Air Quality model and predicted avoided health impacts and related economic values. We also quantitatively assessed the distribution of these benefits by population‐level socioeconomic status. Across the study area, decommissioning the power plants would result in 2 (95% CI: 1–3) avoided premature deaths each year due to reduced PM_2.5_ exposures and greater reductions in hospitalizations and other morbidities. Health benefits resulting from the modeled shutdowns were greatest in areas with lower educational attainment and other economic indicators. Our results suggest that decommissioning these power plants and replacing them with zero‐emissions sources could have broad public health benefits for residents of Colorado, with larger benefits for those that are socially disadvantaged. Our results also suggested that researchers and decision makers need to consider the unique demographics of their study areas to ensure that important opportunities to reduce health disparities associated with point‐source pollution.

## Key Points

- Coal‐fired power plants in Colorado contribute to poor air quality, but two of the largest plants are slated to be decommissioned by 2035
- Retiring these plants now would result in ~40 fewer deaths and $274M in avoided impacts over the remaining 19‐year lifespan of the plants
- Reduced emissions would predominantly benefit low‐income communities which often bear the heaviest environmental burdens

## 1 Introduction

The passage of the Clean Air Act of 1970 has been associated with declines in ambient air pollution in the United States (U.S. Environmental Protection Agency (US EPA), 2017), and these declines have been associated with longer life expectancy (Correia et al., 2013), improved lung function among children (Gauderman et al., 2015), and fewer hospitalizations for respiratory and cardiovascular diseases, among other benefits (U.S. EPA, 2017). However, current levels of PM_2.5_ and O_3_ still pose a public health risk (Gakidou et al., 2017; Lelieveld et al., 2015). Exposures to ambient fine particulate matter (aerodynamic diameter < 2.5 μm; PM_2.5_) and ozone (O_3_) result in approximately 135,000 premature deaths each year in the United States (Fann et al., 2012). Approximately 26% of the years of life lost in 2005 the United States attributable to PM_2.5_ from industrial facility emissions were due to electricity‐generating stations, largely a result of the U.S.'s reliance on coal as an energy source; this percentage decreased to 14% by 2016 (Fann et al., 2013).

Throughout the United States, lower income populations are more likely to live near industrial facilities such as coal‐fired power plants (Collins et al., 2016; Mohai et al., 2009; Mohai & Saha, 2015; Schulz et al., 2016). Exposure to environmental pollutants generated by these industrial sources, including ambient air pollution, is inequitably distributed by class and race (Ash et al., 2013; Brown, 1995; Pastor et al., 2005; Zwickl et al., 2014) and may contribute to differences in health outcomes (Adler & Newman, 2002; Apelberg et al., 2005; Collins et al., 2011; Morello‐Frosch & Jesdale, 2006; Rice et al., 2014). These disparities are in part driven by differing patterns of consumption and exposure; for example, consumption of electricity generated at coal‐fired power plants by White Americans disproportionately exposes Black and Hispanic Americans to air pollutant levels higher than their own consumption generates (Tessum et al., 2019). Several studies have found significant associations between higher ambient air pollution concentrations and lower community‐level SES (Jerrett et al., 2001; Miranda et al., 2011; Morello‐Frosch et al., 2011; Pearce et al., 2006), though this relationship has not been consistently reported across the literature (New York City Department of Health and Mental Hygiene, 2013; Vrijheid et al., 2012). In addition to higher levels of pollution, low SES communities often have unequal access to resources (e.g., heath care or an ability to modify their homes) to help mitigate the negative effects of comparatively higher pollution exposures (Schulz & Northridge, 2004). However, through policy and regulatory mechanisms, ambient air pollution is considered a modifiable risk factor for disease. Environmental policies such as emissions reduction strategies have resulted in decreased population exposure to ambient air pollution (Chan et al., 2012; National Research Council (NRC), 2004), which in turn, has been linked to improved population health outcomes (Gauderman et al., 2015; Lepeule et al., 2012). However, questions remain regarding the relative benefit of these policies to low versus high SES groups (Cesaroni et al., 2013; Levy et al., 2002).

In response to a proposed scenario to retire two coal‐fired power plants in Colorado, we conducted a health impact assessment (HIA) to quantify the likely health benefits of retiring these two generating stations. The study domain is a region with both urban and rural populations that remains understudied with respect to the health effects of ambient air pollution. Our HIA had two objectives. First, we aimed to quantify the health benefits (as avoided deaths, hospitalizations, and other health outcomes) due to reduced air pollutant exposures that would result from decommissioning these two Front Range power plants. Second, we assessed the environmental justice implications for reducing exposures and adverse health effects in the study area.

## 2 Materials and Methods

### 2.1 Study Area and Unit of Analysis

The southern Front Range of Colorado is home to two large coal‐fired power plants: Comanche Generating Station, a 1,410 MW facility located in Pueblo, CO, owned by Xcel Energy and Martin Drake Power Station, a 185 MW facility located in Colorado Springs, CO, owned by Colorado Springs Utility. Comanche Generating Station, the largest in Colorado, operates three units, each with a capacity between 325 and 750 MW. The facility has operated since the 1970s and burns low‐sulfur coal as its primary fuel source (Xcel Energy, 2017a). On 6 June 2018, Xcel Energy released its updated 2016 Electric Resource Plan 120‐Day Report outlining a proposal to shutter Comanche Units 1 and 2 by 2022 and 2025, respectively, and replace this generation source with renewable energy from wind and solar sources (Colorado Public Utilities Commission, 2018; Xcel Energy, 2017b). Martin Drake Power Station currently operates two units, and in 2016, the facility installed flue gas desulfurization scrubbers to reduce sulfur dioxide emissions from the facility (Colorado Springs Utility, 2018; Stanton Anleu, 2016). In 2015, the Utilities Board in Colorado Springs voted to fully decommission the plant by 2035; the process of decommissioning the facility began in 2015 with the shutdown of Unit 5, the facilities' smallest and oldest boiler (Colorado Springs Utility, 2018).

The study area encompassed the entire state of Colorado and included the 5.2 million people who lived in the state in 2014. The Front Range of the Rocky Mountains has a unique combination of topography, meteorology, and sources resulting in a complex air pollutant mix (Vedal et al., 2009). The region is currently designated as a moderate nonattainment area relative to the 2008 ozone National Ambient Air Quality Standard (NAAQS; 75 ppb; U.S. Environmental Protection Agency (US EPA), 2018). As the state has likely missed the 2018 deadline to comply with the 2008 standard, the region will likely receive a “serious nonattainment” designation in 2019 (Colorado Department of Public Health & Environment, 2017).

We chose ZIP code tabulation areas (ZCTA) as the spatial unit of analysis for the Health Impact Assessment. ZCTAs are spatial units designed by the U.S. Census Bureau to closely align with U.S. Postal Service ZIP codes, though there may be some differences in unit boundaries from year to year (Grubesic & Matisziw, 2006). ZCTAs were small enough (median (IQR) area: 252 (34–698) km^2^) to reasonably capture the spatial variability in pollutant exposures across the study area and contained populations large enough (median (IQR) population: 2,584 (472–15,814) persons) to calculate stable hospitalization and mortality rates used in the health impact functions (described below).

**Figure 1:**
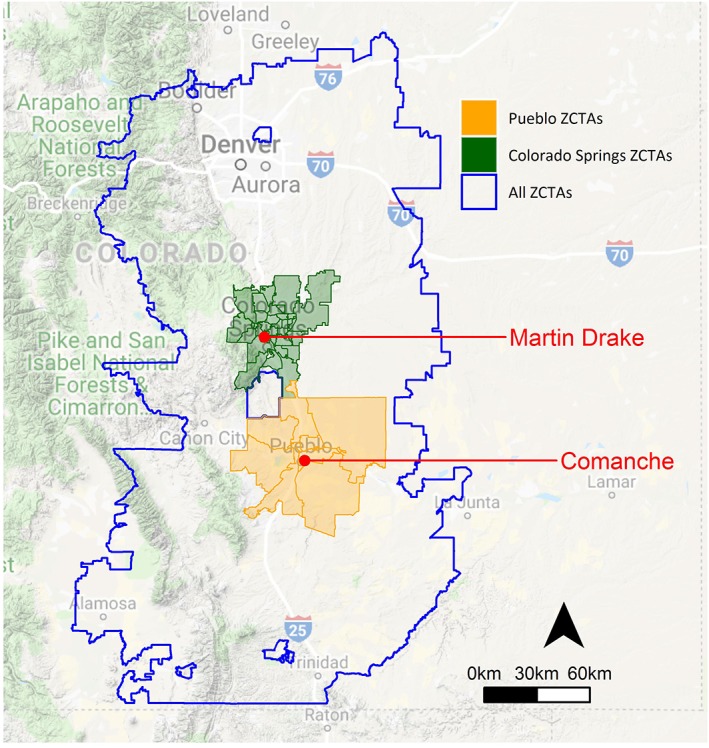
Map of the subset of the study area showing the location of the power plants and the included ZCTAs in the southern Front Range region of Colorado. ZTAs = ZIP Code Tabulation Areas.

Because of local interest in the health effects of the power plants, we also considered a subset of the area consisting of more than 56,000 km^2^ on Colorado's southern Front Range that included the cities of Denver, Colorado Springs, and Pueblo and had a population of 3.8 million people in 2014 (Figure 1). This smaller study area includes ZCTAs within 60–200 km of the power plants, which should account for 50%–75% of the health impacts associated with emissions from these facilities (Goodkind et al., 2019). We also defined two additional two subsets of ZCTAs, one surrounding the Comanche plant and another surrounding the Martin Drake plant. We defined surrounding ZCTAs as polygons that were within or adjacent to the city limits of Colorado Springs or Pueblo.

### 2.2 Ambient Air Pollutant Concentration Modeling

We used the Community Multiscale Air Quality (CMAQ) model (version 5.0.2) to simulate changes in the air pollutant concentrations over the study domain. Details about this version of the model can be found in Gantt et al. (2015). This version of CMAQ has been thoroughly evaluated against ambient measurements by Nolte et al. (2015) and offers reasonable predictions of O_3_ and PM_2.5_ in the state of Colorado (Appel et al., 2017). Briefly, the CMAQ model was used to simulate air pollutant concentrations for a representative summer (29 June to 18 August 2011) and a winter period (2 January to 21 February 2011). Due to the heavy computational burden for CMAQ, we modeled a representative summer and winter period, which accounted for the highest exposure seasons for ozone (peaks in the summer) and fine particulate matter (peaks in the summer and winter) in Colorado. We selected 2011 as the baseline year because this was the most recent version for which validated pollutant emissions were available through the National Emissions Inventory (NEI; U.S. Environmental Protection Agency (US EPA), 2012a). Model results for the first sixteen days were ignored to minimize the influence of initial model conditions, and the HIA analysis was performed with model results from 30 summer and winter days. Results generated for each of these months using the health impact assessment models (described next) were scaled up to represent the full year. This assumes that the average of the summer and winter month results are similar to the true average over the year. Our assumption is supported by data from the local monitoring network (see Figure S1 in the supporting information). For both the full state and the southern Front Range region, levels of PM_2.5_ remained fairly flat throughout the year; ozone displayed the known seasonal patterns of peaks in the summer and lows in the winter. Thus, we were confident that our 2 months in summer and winter would capture the mean exposures across the modeled year.

Model simulations were run at three different resolutions: 36, 12, and 4 km. The 36‐km domain approximately covered the continental United States, the 12‐km domain covered the western United States, and the 4‐km version covered the states of Colorado and Utah and parts of Wyoming, Idaho, New Mexico, and Arizona (Figure S3). The 36‐ and 12‐km model results are used to inform the initial and boundary conditions for the 4‐km version (299 rows × 281 columns × 25 vertical layers). Atmospheric chemistry was simulated using the Carbon Bond 2005 chemical mechanism and particle chemistry and thermodynamics was simulated using the Aerosols Module 5 (Sarwar et al., 2012). As described earlier, anthropogenic emissions were based on the most recent NEI product while biogenic emissions were based on the Biogenic Emission Inventory version 3.14 model (Carlton & Baker, 2011). Meteorological inputs were generated using version 3.1 of the Weather Research and Forecasting model (Skamarock et al., 2008). The CMAQ modeling system that includes the chemical transport model and its relevant inputs for the year 2011 (e.g., meteorology, emissions, and land use types) were requested from the Intermountain West Data Warehouse (Intermountain West Data Warehouse (IWDW), 2018).

### 2.3 Health Impact Assessment Model

A quantitative HIA model was used to predict mortality and morbidity attributable to ambient air pollutant exposures in the study area. We estimated avoided health impacts attributable to changes in O_3_ and PM_2.5_ as these pollutants account for the majority of adverse health outcomes due to ambient air pollutant exposures. Further, concentration‐response functions are readily available for these pollutants (Fann et al., 2012; U.S. Environmental Protection Agency (US EPA), 2012b, U.S. Environmental Protection Agency (US EPA), 2015b). We used area‐specific data from the Colorado Department of Public Health and Environment and Colorado Hospital Association to estimate avoided premature deaths (all causes and nonaccidental for PM_2.5_ and ozone, respectively) and cardiovascular and respiratory hospitalizations resulting from changes in exposure. Because area‐specific data for other important air pollution‐related outcomes, including emergency department visits for asthma, asthma symptom days, and missed days of work or school, were not available, we followed methods described in the U.S. EPA Benefits Mapping and Assessment (BenMAP) tool (U.S. Environmental Protection Agency (US EPA), 2016b) and used national incidence rates to estimate benefits in the study domain.

The HIA model implemented in this report was based on methods used by BenMAP (U.S. Environmental Protection Agency (US EPA), 2016b). The health impact function (equation (1)), which generates estimates of the number of attributable (or avoided) health impacts due to a change in exposure, is adapted from the expression for attributable risk and is defined as follows:

(1) $$∆Y=y_{0}\times \left(1-e^{-\beta \;∆x}\right)\times P\times D$$

where Δ*Y* is the change in the number of attributable impacts during the study period (cases), *y*_0_ is the baseline health outcome incidence rate (cases per person per day), *β* is the concentration‐response (CR) coefficient (1/ppb or 1/μg/m^−3^), Δ*x* is the estimated change in daily mean ambient concentration (ppb or μg/m^−3^), *P* is the number of people exposed, and *D* is the number of days in the study period (U.S. Environmental Protection Agency (US EPA), 2015a).

We modeled two health benefit scenarios in our HIA (Table 1). Health Benefit Scenarios 1 and 2 both assessed the health benefits of shutting down the Martin Drake facility and the two units at Comanche at the same time. In Health Benefits Scenario 1, health benefits were estimated for the change in exposure relative to the 2016 counterfactual baseline in which the 2011 SO_2_ emissions for Martin Drake boilers were reduced by 96.7% to account for a flue gas desulfurization system installed that year, and in Health Benefits Scenario 2, health benefits were estimated for the change in exposure relative to the 2011 baseline. It was necessary to run two separate scenarios to account for the decrease in emissions at Martin Drake between when the emissions inventory was developed and today to avoid overestimating the future benefits of decommissioning the plants. It is important to note that emissions at all other facilities included in the emissions inventory remain the same in both scenarios to quantify specific changes in air quality due to changes in emissions from the Martin Drake and Comanche facilities.

**Table 1:** Summary of the Health Benefits Scenarios Used in This HIA

Scenario name	Baseline exposure scenario	Post‐shutdown exposures
Health Benefits Scenario 1	Model Run 1: CMAQ was run for representative summer and winter periods using the 2011 NEI for emissions at all sources in the area except Martin Drake. For Martin Drake, we adjusted the 2011 NEI emissions to account for scrubbers installed in 2016.	Model Run 3: CMAQ was run for representative summer and winter periods using the 2011 NEI for emissions at all sources in the area except Comanche Units 1 and 2 and Martin Drake Units 5, 7, and 8, which were “zeroed out.”
Health Benefits Scenario 2	Model Run 2: CMAQ was run for representative summer and winter periods using the 2011 NEI for emissions at all sources in the area without adjustments for the scrubbers installed at Martin Drake.	Model Run 3: CMAQ was run for representative summer and winter periods using the 2011 NEI for emissions at all sources in the area except Comanche Units 1 and 2 and Martin Drake Units 5, 7, and 8, which were “zeroed out.”

Additional details on the health impact function inputs, including the baseline health outcome incidence rates, the CR coefficients, the exposure assessment methodology, and the identification of exposed populations, are included in the supporting information.

We accounted for uncertainty in each of the health impact model inputs using a Monte Carlo simulation. For each impact function input, including the change in exposure, health rates, concentration‐response coefficient, and population, we generated a normal distribution of possible values using the mean estimate and the standard error of the mean. Further details on how we calculated each of the HIA inputs is found in the supporting information. Briefly, uncertainty in the change in exposure was based on the standard error of the population‐weighted means calculated for each ZCTA. We needed to convert the 4 km × 4 km CMAQ grid estimates of changes in air pollutant concentrations to population‐weighted concentrations at the ZCTA level because the health outcome rates and EJ indicators could not be extracted to the CMAQ grid. For health rates, we used the standard error of the rate calculated using a Poisson regression model with no covariates. For the concentration‐response coefficient, uncertainty in the coefficient was based on the standard error of the pooled effect estimate from the meta‐analysis. Lastly, uncertainty in population estimates for each ZCTA and age group were based on standard errors calculated from the margins of error reported in the American Community Survey (U.S. Census Bureau, 2018b). For each input, we assumed a normal distribution around the mean. For the exposure data, we based these parameters on empirical data. However, for the CR, population, and baseline health status, we were working with secondary data and did not have access to the underlying population parameters. As such, we used *n* = 1,000 random draws with replacement from these distributions in our health impact function. From these *n* = 1,000 iterations, we estimated the median, 2.5th, and 97.5th percentiles for avoided impacts. We reported health benefits on an annual basis and aggregated over the remaining lifespan of the facilities, calculated using the planned 2035 shutdown for Martin Drake and the baseline year for the CMAQ simulation (2016 for Health Benefits Scenario 1 or 2011 for Health Benefits Scenario 2).

### 2.4 Economic Analysis

We monetized the health benefits of each shutdown scenario using values assigned to each outcome by U.S. Environmental Protection Agency (US EPA) (2010). For this HIA, we used the same monetized values used in the most recent Regulatory Impact Analysis for the ozone NAAQS (U.S. Environmental Protection Agency (US EPA), 2015b). All monetized impacts are reported in 2011 dollars projected to a 2024 income level following methods reported by U.S. EPA. For mortality, we used the willingness to pay estimate of $10 million per death (U.S. Environmental Protection Agency (US EPA), 2010, 2015b). For hospitalizations and other health‐care utilization outcomes (e.g., emergency department visits), the value assigned to each hospitalization avoided was based on the average cost of care, also taken from the ozone Regulatory Impact Analysis (U.S. Environmental Protection Agency (US EPA), 2015b). For outcomes such as work loss days, school loss days, or asthma exacerbation days, values were based on the potential lost wages of the employee or adult who misses work to care for a sick child (U.S. Environmental Protection Agency (US EPA), 2015b). To account for the time‐value of money, we applied a 3% discount rate to the accrued monetized health benefits following methods recommended by U.S. Environmental Protection Agency (US EPA) (2010).

### 2.5 Environmental Justice Analysis

We relied on the U.S. EPA definition of environmental justice in order to interpret the results of our health impact assessment. U.S. EPA defines environmental justice as “the fair treatment and meaningful involvement of all people regardless of race, color, national origin, or income with respect to the development, implementation, and enforcement of environmental laws, regulations, policies” (U.S. Environmental Protection Agency (US EPA), 2016a, p. 1) and specifies that fair treatment under environmental policies extends to the distribution of benefits resulting from those policies.

We used two approaches to examine whether decommissioning the two generating stations had an equal benefit to all residents, and if not, whether environmental justice communities (e.g., ZCTAs with higher proportions of persons of color and/or low‐income communities) received fair treatment under the shutdown scenarios. For both approaches described here, we focused on avoided premature deaths and hospitalizations as spatially resolved baseline data were available for these outcomes. First, changes in exposure (Δ ppb or Δ μg/m^−3^) and health benefit rates (avoided deaths or hospitalizations per 10,000 people) were mapped for each pollutant and health outcome, which helps to identify areas within the study area that benefit most under the shutdown scenarios. Following this mapping step, differences in health benefits across demographic and socioeconomic groups were assessed using the concentration curve. The concentration curve is a graphical representation of inequality that first ranks ZCTAs by their degree of social advantage and then compares the distribution of benefits against this social advantage ranking (Harper et al., 2013). Because the concentration curves are sensitive to the study boundaries (Martenies et al., 2017), we focused on inequality within the southern Front Range region of the state, as this area is more densely populated than the state as a whole.

Following other HIAs examining environmental justice impacts, we selected the percentage of the ZCTA population that is non‐Hispanic White (NHW) and median ZCTA‐level income to serve as proxies for social advantage (Fann et al., 2011; Harper et al., 2013; Martenies et al., 2017; Wesson et al., 2010). Because of our interest in understanding patterns of benefits across SES groups, we also included the percentage of the civilian workforce that is employed, the percentage of the population > age 25 to have a high school diploma (or equivalent), the percentage of households that speak proficient English, and the percentage of households with income above the poverty level as alternative indicators of social advantage at the ZCTA level. There are no established standards against which to compare the concentration curve. In general, we interpreted a concentration curve above the 1:1 line to mean that benefits (i.e., avoided health impacts) were higher among ZCTAs with lower social advantage (Maguire & Sheriff, 2011).

## 3 Results

### 3.1 Reductions in Ambient Exposures to PM_2.5_ and O_3_

Exposures generally decreased when emissions at the generating stations were reduced (Table 2). For PM_2.5_, decreases in the seasonal and daily means were greatest for the summer months; on average, the seasonal mean PM_2.5_ concentration during the summer across all ZCTAs decreased 0.006 μg/m^3^ (SD = 0.01 μg/m^3^). Similarly, for ozone, reductions in exposure at the ZCTA level were strongest in the summer, with an average decrease in ozone of 0.025 ppbv. This was expected since photochemical production of ozone is largest in the summer. Increases in winter seasonal mean and daily mean ozone concentrations were likely the result of alterations in the overall NO_*x*_‐O_3_ relationship that occurred when combustion at the plants was reduced. NO_*x*_ emissions generated by the plant contribute to titration of ground‐level ozone (i.e., NO reacts with O_3_ to produce O_2_ and NO_2_ (Song et al., 2011)); reduced combustion at the plant resulted in lower atmospheric NO concentrations and decreased ozone scavenging. Reductions in exposure for Health Benefits Scenario 2, which used the 2011 emissions at Martin Drake, were larger in magnitude than for Health Benefits Scenario 1, though the patterns across seasons were similar (Table S2).

**Table 2:** Summary Statistics for the Change in Population‐Weighted Exposures to PM_2.5_ (μg/m^3^) and ozone (ppbv) Across All ZCTAs in Colorado for Health Benefits Scenario 1, Which Compared Concentrations Based on a Counterfactual 2016 Baseline Emissions to a Full Shutdown at Martin Drake and Partial Shutdown at Comanche

Pollutant	Season	Metric	Mean (SD)	Min	Median	Max
PM_2.5_	Winter	Monthly mean	−0.005 (0.010)	−0.082	−0.001	0.000
	Winter	Daily mean	−0.004 (0.015)	−0.259	0.000	0.015
	Summer	Monthly mean	−0.006 (0.012)	−0.098	−0.003	0.000
	Summer	Daily mean	−0.006 (0.015)	−0.259	−0.001	0.006
O_3_	Winter	Monthly mean	0.017 (0.036)	−0.003	0.003	0.201
	Winter	Daily mean	0.017 (0.068)	−0.131	0.000	1.115
	Winter	Daily 1‐hr max	−0.011 (0.046)	−0.645	0.000	0.316
	Winter	Daily 8‐hr max	−0.003 (0.024)	−0.375	0.000	0.381
	Summer	Monthly mean	−0.025 (0.033)	−0.111	−0.027	0.226
	Summer	Daily mean	−0.024 (0.080)	−0.696	−0.006	1.214
	Summer	Daily 1‐hr max	−0.182 (0.351)	−5.248	−0.049	0.140
	Summer	Daily 8‐hr max	−0.088 (0.155)	−1.876	−0.030	0.140

*Note*. SD = standard deviation

**Figure 2:**
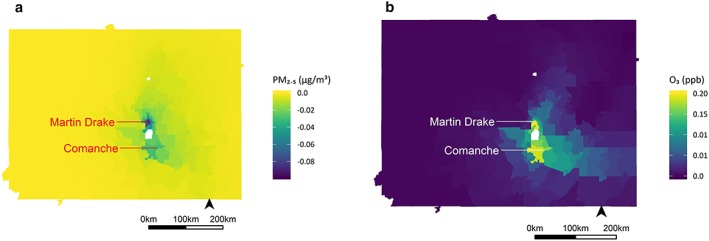
Changes in mean summer PM_2.5_ (a; μg/m^3^) and O_3_ (b; ppb) concentrations at the ZIP Code Tabulation Area level for Health Benefits Scenario 1.

Changes in exposures occurred were greatest near the power plants. Figure 2 shows the change in summer PM_2.5_ (A) and ozone (B) concentrations at the ZCTA level for Scenario 1. (Comparable maps of the changes in exposure during the winter and for summer and winter changes in exposure for Scenario 2 are included in the supporting information; Figures S4 and S5). For PM_2.5_, concentrations near the power plants were reduced, with lower reductions farther from the plants as the emissions were diluted with transport; for O_3_, concentrations near the plant increased slightly under the emissions reduction scenario and decreased slightly elsewhere across the study area. These changes in PM_2.5_ and O_3_ were small relative to the baseline levels of each pollutant. (Figure S6 shows the ratios of ΔPM_2.5_/PM_2.5_ at baseline and ΔO_3_/O_3_ at baseline for the summer and winter seasons. The highest ratios are seen within 150 km of the power plants, similar to the patterns observed for the changes in pollutant concentrations.)

### 3.2 Estimated Air Pollution‐Related Health Benefits of Decommissioning the Power Plants

Retiring both plants would have modest health benefits for residents of the southern Front Range in Colorado (Table 3). For Scenario 1, we estimated that reducing population exposures to PM_2.5_ and O_3_ would result in 2 (95% CI: 1–3) and <1 (95% CI: 0–1) fewer premature deaths each year, respectively, across the state (Table 3). Over the course of the remaining lifespan of these power plants, this would result in approximately 38 (19–57) avoided deaths in the region. Among the largest accrued health benefits were avoided asthma symptom days among children (38 (95% CI: −320–840) due to PM_2.5_ and 190 (95% CI: −5,300–15,000) due to O_3_) and minor restricted activity days among adults (530 (95% CI: −38–3700) due to PM_2.5_ and 1,000 (95% CI: −320–11,000) due to O_3_). We also estimated that children in the study area would miss a total of 60 (95% CI: −54–970) fewer days of school each year due to lower O_3_ exposures. The ZCTAs within and adjacent to the city of Colorado Springs received the largest proportion of the health benefits (Table 3), coinciding with the greater reductions in PM_2.5_ as a result of decommissioning the plants (Figure 2) and greater ZCTA populations.

**Table 3:** Summary of Annual and Accrued Health Benefits (through 2035) as the Median Number of Avoided Premature Deaths and Cases of Morbidity (2.5th to 97.5th Percentiles) Across the Study Area and for Each Subsection of the Study Area for Health Benefits Scenario 1

		All ZCTAs	SFR	Colorado Springs	Pueblo
	Outcome	Benefits per year			
PM_2.5_	AC mortality	2 (1, 3)	1 (1, 2)	1 (1, 1)	0 (0, 0)
	CVD hospitalization	0 (0, 1)	0 (0, 1)	0 (0, 0)	0 (0, 0)
	RES hospitalization	0 (0, 1)	0 (0, 1)	0 (0, 0)	0 (0, 0)
	ED visit for asthma	0 (0, 0)	0 (0, 0)	0 (0, 0)	0 (0, 0)
	AST symptom day	2 (−17, 44)	1 (−11, 27)	1 (−8, 20)	0 (−2, 5)
	MRAD	28 (−2, 200)	23 (2, 100)	19 (2, 74)	3 (0, 17)
	Work loss day	5 (0, 32)	4 (0, 17)	3 (0, 12)	1 (0, 3)
O_3_	NA mortality	0 (0, 1)	0 (0, 0)	0 (0, 0)	0 (0, 0)
	RES hospitalization	1 (−11, 22)	0 (−9, 6)	0 (−5, 3)	0 (−4, 2)
	ED visit for asthma	0 (0,0)	0 (0, 0)	0 (0, 0)	0 (0, 0)
	AST symptom day	10 (−220, 790)	6 (−110, 270)	4 (−63, 170)	1 (−28, 57)
	MRAD	53 (−17, 590)	23 (−15, 170)	14 (−7, 100)	6 (−6, 34)
	School absence day	60 (−54, 970)	29 (−35, 300)	18 (−17, 170)	7 (−13, 62)
				
		Accrued benefits through 2035			
PM_2.5_	AC mortality	38 (19, 57)	19 (9, 38)	19 (9, 38)	0 (0, 0)
	CVD hospitalization	0 (0, 19)	0 (0, 19)	0 (0, 0)	0 (0, 0)
	RES hospitalization	0 (0, 19)	0 (0, 19)	0 (0, 0)	0 (0, 0)
	ED visit for asthma	0 (0, 0)	0 (0, 0)	0 (0, 0)	0 (0, 0)
	AST symptom day	38 (−320, 840)	19 (−210, 510)	19 (−152, 380)	0 (−38, 95)
	MRAD	530 (−38, 3,700)	440 (38, 1,900)	360 (38, 1,400)	57 (0, 323)
	Work loss day	95 (0, 610)	76 (0, 320)	57 (0, 230)	19 (0, 57)
O_3_	NA mortality	0 (0, 19)	0 (0, 0)	0 (0, 0)	0 (0, 0)
	RES hospitalization	19 (−210, 420)	0 (−170, 110)	0 (−95, 57)	0 (−76, 38)
	ED visit for asthma	0 (0, 0)	0 (0, 0)	0 (0, 0)	0 (0, 0)
	AST symptom day	190 (−5,300, 15,000)	110 (−2,100, 5,100)	76 (−1,200, 3,100)	19 (−530, 1,100)
	MRAD	1,000 (−320, 11,000)	440 (−290, 3,300)	270 (−130, 2,000)	110 (−110, 650)
	School absence day	1,100 (−1,000, 18,000)	550 (−670, 5,700)	340 (−320, 3,500)	130 (−250, 1,200)

*Note*. Values have been rounded to two significant figures. AC = all‐cause; AST = asthma; ED = emergency department; MRAD = minor‐restricted activity day; NA = non accidental; RES = respiratory; SFR = Southern Front Range.

Health benefits were larger for Health Benefits Scenario 2 due to the greater change in exposure concentration when not accounting for the control technologies installed at Martin Drake in 2016 (Table S3). For example, we estimated that reducing population exposures to PM_2.5_ and O_3_ would result in 5 (95% CI: 3–7) and <1 (95% CI: 0–1) fewer premature deaths each year, respectively, under Scenario 2. Other outcomes were similarly increased.

For health impacts attributable to PM_2.5_ exposures, most avoided impacts occurred in close proximity (i.e., within 60 km) to the generating stations (Figure S7). Figure S7 shows the rates of avoided premature deaths due to PM_2.5_ (A) and O_3_ (B). (Comparable figures for Health Benefits Scenario 2 are found in the supporting information; Figure S8.) The spatial trends in avoided health impacts mirrors the spatial patterns for decreases in PM_2.5_ resulting from plant shutdowns (Figure 2).

### 3.3 Monetized Benefits of Decommissioning the Power Plants

Decommissioning the power plants resulted in a substantial monetized health benefit to the southern Front Range of Colorado. The total monetized value of the health benefits of retiring the oldest coal‐fired boilers at Martin Drake and Comanche was approximately $270 million (95% CI: $130–$510 million) over the remaining life span of the power plants for Health Benefits Scenario 1 (assuming a discount rate of 3%; Table 4). This result was driven by the four estimated avoided premature deaths per year, which were valued at $10 million per premature death. Again, the ZCTAs within and adjacent to Colorado Springs received about half the monetized benefits of the reduction in emissions.

**Table 4:** Summary of Total Monetized Value of Annual and Accrued Health Benefits (Through 2035) in $1,000s (2.5th to 97.5th Percentiles) Across the Study Area and for Each Subsection of the Study Area for Health Benefits Scenario 1

		All ZCTAs	SFR	Colorado Springs	Pueblo
	Outcome	Monetized benefits per year			
PM_2.5_	AC mortality	17,000 (10,000, 26,000)	13,000 (7,700, 20,000)	9,400 (5,500, 14,000)	2,800 (1,700, 4,000)
	CVD hospitalization	7 (0, 46)	6 (0, 26)	4 (0, 17)	1 (0, 7)
	RES hospitalization	4 (‐1, 39)	4 (0, 22)	3 (0, 15)	1 (0, 6)
	ED visit for asthma	0 (0, 0)	0 (0, 0)	0 (0, 0)	0 (0, 0)
	AST symptom day	0 (−1, 3)	0 (−1, 2)	0 (−1, 1)	0 (0, 0)
	MRAD	2 (0, 13)	2 (0, 7)	1 (0, 5)	0 (0, 1)
	Work loss day	1 (0, 5)	1 (0, 3)	0 (0, 2)	0 (0, 0)
O_3_	NA mortality	440 (−1,600, 5,800)	180 (−1,400, 2,200)	54 (−760, 1,100)	83 (−520, 710)
	RES hospitalization	24 (−400, 830)	−4 (−340, 240)	−8 (−180, 110)	2 (−150, 82)
	ED visit for asthma	0 (0, 0)	0 (0, 0)	0 (0, 0)	0 (0, 0)
	AST symptom day	1 (−17, 47)	0 (−7, 16)	0 (−4, 10)	0 (−2, 3)
	MRAD	4 (−1, 40)	2 (−1, 12)	1 (0, 7)	0 (0, 2)
	School absence day	6 (−5, 95)	3 (−3, 30)	2 (−2, 18)	1 (−1, 6)
		Accrued monetized benefits through 2035			
PM_2.5_	AC mortality	270,000 (160,000, 400,000)	200,000 (120,000, 300,000)	140,000 (85,000, 220,000)	43,000 (27,000, 62,000)
	CVD hospitalization	100 (−4, 710)	88 (8, 400)	68 (7, 260)	18 (0, 100)
	RES hospitalization	68 (−13, 600)	58 (−4, 340)	44 (−3, 220)	13 (−1, 98)
	ED visit for asthma	0 (0, 0)	0 (0, 0)	0 (0, 0)	0 (0, 0)
	AST symptom day	1 (−16, 41)	1 (−10, 25)	1 (−8, 18)	0 (−2, 4)
	MRAD	29 (−2, 200)	24 (2, 110)	20 (2, 77)	3 (0, 18)
	Work loss day	11 (−1, 74)	9 (1, 39)	7 (1, 28)	1 (0, 7)
O_3_	NA mortality	6,700 (−25,000, 90,000)	2,700 (−21,000, 34,000)	830 (−12,000, 16,000)	1,300 (−8,000, 11,000)
	RES hospitalization	370 (−6,200, 13,000)	−61 (−5,300, 3,600)	−130 (−2,700, 1,700)	27 (−2,300, 1300)
	ED visit for asthma	0 (0, 2)	0 (0, 1)	0 (0, 0)	0 (0, 0)
	AST symptom day	9 (−260, 720)	5 (−100, 250)	3 (−58, 150)	1 (−26, 52)
	MRAD	56 (−18, 610)	24 (−15, 180)	15 (−7, 110)	6 (−6, 36)
	School absence day	90 (−81, 1,500)	43 (−52, 450)	27 (−26, 280)	11 (−19, 93)
	Total	270,000 (130,000, 510,000)	200,000 (91,000, 340,000)	140,000 (70,000, 240,000)	44,000 (16,00, 74,000)

*Note*. Monetized values are reported as 2011$ projected to a 2024 income level following methods reported by U.S. EPA (U.S. Environmental Protection Agency [US EPA], 2015a). Accrued benefits are discounted at a 3% rate. Values have been rounded to two significant figures. AC = all‐cause; AST = asthma; ED = emergency department; MRAD = minor‐restricted activity day; NA = nonaccidental; RES = respiratory.

The monetized value of annual health benefits for Health Benefits Scenario 2 were higher (Table S4), but the relative contributions of avoided premature deaths to the total monetized value remained similar.

### 3.4 Environmental Justice Implications of Decommissioning the Power Plants

**Figure 3:**
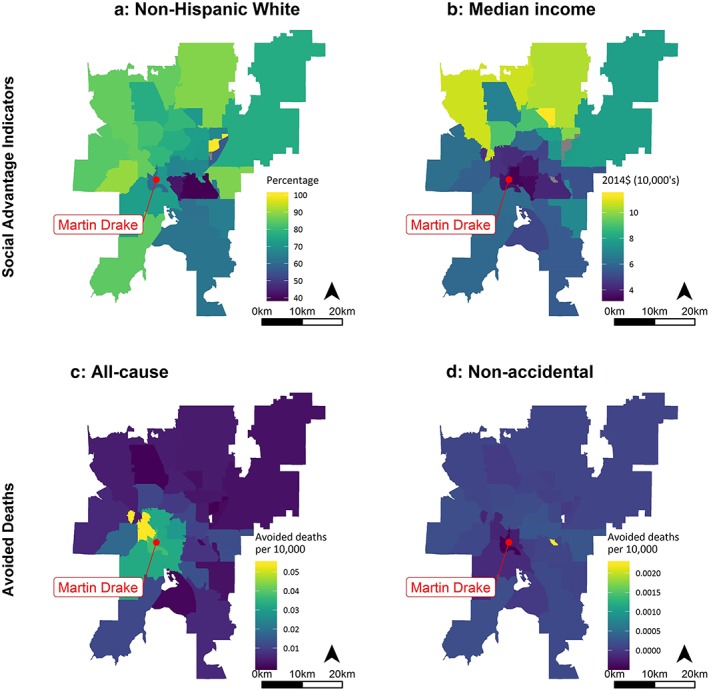
The percentage of ZIP Code Tabulation Area populations that are non‐Hispanic White (a) and median income (b) and benefits of reducing emission (Health Benefits Scenario 1) as avoided deaths attributable to PM_2.5_ (c) and O_3_ (d) ZCTAs near the Martin Drake power plant in Colorado Springs, CO.

**Figure 4:**
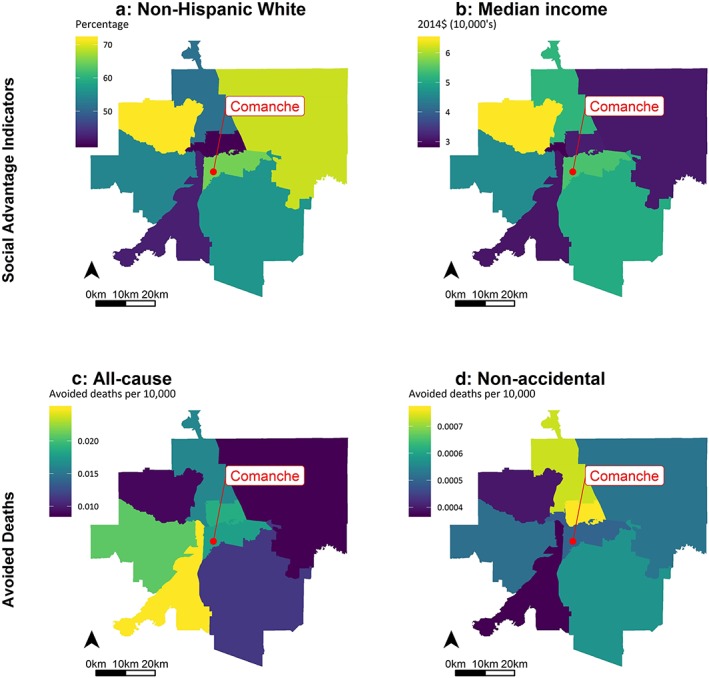
The percentage of ZIP Code Tabulation Area populations that are non‐Hispanic White (a) and median income (b) and annual benefits of reducing emission (Health Benefits Scenario 1) as avoided deaths attributable to PM_2.5_ (c) and O_3_ (d) ZCTAs near the Comanche power plant in Pueblo, CO.

Many of the communities closest to Comanche and Martin Drake have higher proportions of residents of color and lower median incomes relative to more distant ZCTAs (Figure 3). In the southern Front Range region, median incomes varied more than the percentage of the population that was NHW in each ZCTA. Median incomes in the Southern Front Range ranged from $15,469 to $129,266, with a mean of $62,424 and a coefficient of variation (CV) of 37.3%; comparatively, the percentage of NHW residents ranged from 13.6% to 100%, with a mean of 73.0% and a CV of 26.6%. Figures 3 and 4 show the median income and percentage of the population that is NHW in ZCTAs within and adjacent to the municipal boundaries of Colorado Springs (Figures 3a and 3b) and Pueblo (Figures 4a and 4b). (Maps for the other social advantage indicators are shown in Figure S9.) For both cities, ZCTAs closer to the power plants tended to have higher proportions of persons of color and lower median incomes.

For both health scenarios, the greatest health benefit rates (e.g., avoided deaths per 10,000 residents) occurred in ZCTAs with lower median incomes and higher proportions of non‐NHW residents (Figures 3c and 3d and 4c and 4d).

**Figure 5:**
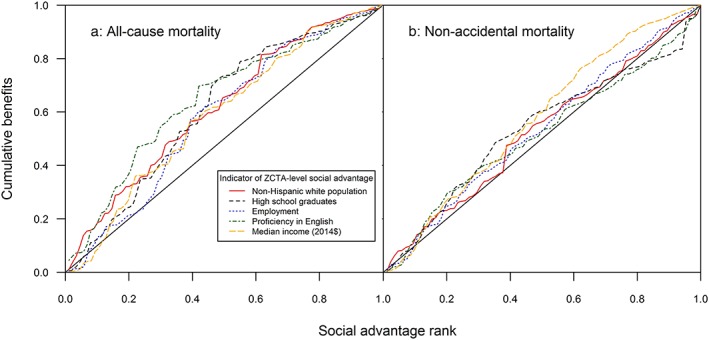
Concentration curves for rates of avoided deaths (per 10,000 persons) resulting from reduced PM_2.5_ (a) or O_3_ (b) exposures under Health Benefits Scenario 1 when ranking ZCTAs by indicators of social advantage. ZTAs = ZIP Code Tabulation Areas.

The concentration curves, which measured the degree of inequality in health benefits by social advantage, indicated inequality in distribution of health benefits (assessed as avoided deaths or hospitalizations per 10,000 people) based on ZCTA‐level median income, educational attainment, and civil workforce employment. Figure 5 shows the concentration curves for rates of avoided deaths (all cause and nonaccidental) attributable to PM_2.5_ (A) and O_3_ (B) when ranking populations by each of the indicators for Health Benefits Scenario 1 for all ZCTAs in the southern Front Range region. (Similar curves for hospitalizations are shown in Figure S10 of the supporting information.) Figure 5 demonstrated that the CI curves for PM_2.5_ impacts were above the 1:1 line for all indicators of race/ethnicity and SES, but the degree of inequality was strongest when using education and household proficiency in English compared to the traditional indicators of race/ethnicity and median income. These results suggested that there was less advantage in terms of health benefits for populations with higher proportions of residents of color (nor do they receive a disproportionately low number of benefits) and that ZCTAs with lower SES received a higher proportion of health benefits relative to ZCTAs with higher SES (measured by educational attainment level). Because most sections of the concentration curve remained above the 1:1 line (i.e., no increases in impacts occurred at most level of social advantage), the decommissioning of the power plants was considered an equitable policy option when considering communities ranked by socioeconomic status.

## 4 Discussion

We conducted a quantitative HIA to assess the potential health benefits of decommissioning Martin Drake Power Station in Colorado Springs and substantially reducing emissions at Comanche Generating Station in Pueblo, CO, for residents of Colorado's southern Front Range. We found that reducing emissions at these generation stations would result in health benefits for the region, including two avoided premature deaths each year, and that these benefits would largely be concentrated in the ZCTAs closest to the power plants. In our assessment, health benefits were greater for reductions in PM_2.5_ than for reductions in O_3_ due to the more consistent decreases in PM_2.5_ across the modeled periods and the stronger associations between health outcomes and PM_2.5_ exposures. Although Martin Drake is the smaller of the facilities included in this assessment, we found that residents in Colorado Springs living near the plant would incur most of the health benefits. This is largely due to population density in the area near the facility compared to the areas surrounding the Comanche facility in Pueblo, CO. In addition, we found that ZCTAs with lower median incomes, lower educational attainment, and lower percent employed would benefit most from the shutdown of these facilities. Our finding that ZCTAs with lower educational attainment and lower household proficiency in English receive a substantial proportion of the health benefits has potential environmental justice implications for the study area.

Across the United States, coal remains an important fuel source for electricity generation and is responsible for 30.1% (1,208 billion kWh) of the electricity used in 2017 (U.S. Energy Information Administration, 2018a). In comparison, natural gas, wind, and photovoltaic solar were responsible for 31.7% (1,273 billion kWh), 6.3% (254 billion kWh), and 1.2% (53 billion kWh) of electricity generated, respectively (U.S. Energy Information Administration, 2018a). Overall generation capacity is projected to drop by 65 GW between 2018 and 2030 but remain constant between 2030 and 2050 (U.S. Energy Information Administration, 2018b). The two coal‐fired generating stations included in this analysis are scheduled to be replaced by primarily renewable sources in the region and natural gas in a neighboring state. Replacement with noncarbon sources and natural gas should reduce emissions in the region due to lower per kilowatt hour emissions from natural gas compared to coal (U.S. Government Accountability Office, 2012).

The Colorado Front Range region remains understudied with respect to the health effects of air pollution, despite long term nonattainment of the ozone NAAQS (U.S. Environmental Protection Agency (US EPA), 2018) and a unique mix of urban and rural sources of pollution (Vedal et al., 2009). Our HIA demonstrated that small reductions in ambient concentrations can have public health benefits, particularly for the urban areas around Colorado Springs and the more rural areas surrounding Pueblo, CO.

This Health Impact Assessment provides an important baseline for future studies in the area to take advantage of the natural experiment that arises from the shutdown of these facilities. In our analysis, we estimated the potential accrued health benefits of retiring these coal power plants by the year 2035. The Colorado Public Utilities commission recently approved Xcel's plan to shut down the coal‐fired boilers at Comanche between 2022 and 2025, and the Utilities Board of Colorado Springs has decided to shutter Martin Drake by 2035 (Colorado Public Utilities Commission, 2018; Colorado Springs Utility, 2018). Depending on the timeline of these shutdowns, our estimate of accrued benefits may represent the higher end of the range of possible benefits.

Other HIAs and similar studies have also reported differences in the distribution of impacts or benefits relative to population‐level indicators of income, socioeconomic status, or vulnerability to exposures and health outcomes. HIAs that incorporate indicators of population vulnerability—that is, extrinsic factors that result in more severe outcomes after exposures (Sacks et al., 2011)—have demonstrated that populations with higher baseline rates of air‐pollution related diseases tend to receive greater benefits when control technologies are applied to pollution sources, for example, power plants or buses (Levy et al., 2002; Levy et al., 2007; Levy et al., 2009). In a case study of power plant control options in Detroit, MI, researchers found that multipollutant approaches that reduced both PM_2.5_ and ozone concentrations would result in greater health benefits overall than single‐pollutant approaches and would result in greater health benefits for low‐income and non‐White residents (Fann et al., 2011; Wesson et al., 2010). A similar analysis found that control strategies that targeted smaller coal‐burning facilities near residential areas in Detroit also conferred more benefits for highly exposed low‐income populations (Martenies et al., 2018). Our results of increased benefits for residents living in ZCTAs with lower median incomes, educational attainment, and employment are in line these previous studies. Taken together, these studies emphasize there are persistent environmental justice concerns related to ambient air pollution in the United States, particularly from industrial sources such as electricity generating stations.

In contrast to other national and local‐scale HIAs, however, we did not observe substantially greater benefits for residents living in ZCTAs with higher proportions of residents of color. (The curve, though above the 1:1 line, does not extend as far as curves for SES indicators.) This result is likely due to the demographics of Colorado residents and the distribution of these populations across the state. In Colorado, 68.3% of residents identify as NHW, 21.5% identify as Hispanic/Latinx (of any race), and 10% identify as any other racial group (U.S. Census Bureau, 2018a). Median incomes varied more than the percentage of residents of color in each ZCTA in our study area, suggesting race/ethnicity is not a sufficient indicator of population disadvantage when examining localized health benefits within the state. Given the long history of environmental racism in the United States (Brulle & Pellow, 2006), population‐ and individual‐level race or ethnicity will likely be an appropriate indicator in many study areas. However, as demonstrated by our results, focusing on race/ethnicity alone may miss key indicators of environmental inequality or important opportunities to improve environmental justice outcomes for other disproportionately impacted groups. The choice of appropriate indicator may be area specific, which could make comparisons across studies difficult, but may be more useful for local decision makers. For example, studies in New York City have demonstrated that traffic‐related air pollutant exposures tend to be higher for household with higher incomes and neighborhoods with higher SES due to the distribution of populations relative to major roadways in the city (Hajat et al., 2013; Shmool et al., 2015), suggesting indicators other than income are more appropriate in that location. Our results emphasize that decision makers should expand the range of characteristics included in environmental justice assessments, particularly in areas with less racial diversity than the national average.

### 4.1 Limitations

Importantly, the health benefits outline in this report should be considered a subset of the total benefits of decommissioning the two coal‐fired power plants. Our HIA was quantitative in nature and therefore limited to the subset of outcomes for which we have sufficient baseline incidence data and concentration‐response functions (O'Connell & Hurley, 2009). We were only able to include health impacts for which there is sufficient evidence of causality (based on EPA determinations; U.S. Environmental Protection Agency (US EPA), 2009, 2013). There are likely other health benefits resulting from reductions in ambient concentrations of PM_2.5_ and O_3_, including reductions in neurocognitive effects and adverse birth outcomes. For example, retirement of coal‐ and oil‐fired power plants in California has been associated with decreases in the risk of preterm birth for pregnant mothers and increases in fertility for those living within 5 km of a facility (Casey et al., 2018; Casey et al., 2018). Due to this lack of complete benefits assessment, any cost‐benefit analysis based on the monetized impacts would likely underrepresent the true public health and economic benefits of decommissioning the plants.

Similarly, we only considered potential benefits and we unable to account for potential harms or other costs associated with decommissioning the plants. For example, we are unable include health and economic effects of job loss related to the plant closures. Other potential costs to the communities include rate changes associated with increased investment in renewable energy. The distribution of these costs is likely to disproportionately impact some rate payers over others. Additionally, we are not able to incorporate the local effects of climate change or other important environmental impacts related to the reduction of emissions in the region. The benefits reported here should be considered a subset of the total impacts of decommissioning the power plants.

The monetized value of the health benefits (i.e., avoided adverse health impacts) of retiring the coal‐fired power plants are not area specific; instead, we use the value of a statistical life and morbidity cost estimates for the United States as a whole. Value of a statistical life estimates are based on willingness to pay to reduce the risk of dying in the next year and, therefore, are subject to an individual's age, income, and perceptions of health and morality risk (Hammitt, 2000, 2007; Viscusi & Aldy, 2003; U.S. Environmental Protection Agency (US EPA), 2010). Similarly, the value of morbidity outcomes depends on several area‐specific factors, including the cost of healthcare (U.S. Environmental Protection Agency (US EPA), 2010). The monetized impacts presented here, though inherently uncertain, are useful for estimating the magnitude of the potential monetized benefit for the state of Colorado and the cities surrounding the power plants.

We note some additional limitations to this analysis that affect the interpretation of the HIA results. As in most HIAs, the concentration response functions implemented in this analysis were based on previous epidemiologic studies and these original studies may not be fully generalizable to our study area (Hubbell et al., 2009). When available, we have selected concentrations‐response functions from nationally representative studies, for example, the American Cancer Society cohort study is considered to be robust and has been used in other HIAs (Fann et al., 2012; Levy et al., 2002; U.S. Environmental Protection Agency (US EPA), 2012b). However, generalizability to the entire study population may be limited, as the ACS cohort has few non‐White participants, with higher than average income education levels compared to the general population (Krewski et al., 2009). For other outcomes for which national studies were not available, for example, hospitalizations, we pooled concentration response coefficients from multiple studies. This pooling approach help to address differences across study populations and is also widely used in the HIA literature (Hubbell et al., 2009; U.S. Environmental Protection Agency (US EPA), 2012b). When implementing the HIA, we often relied on epidemiology studies that used central‐site monitoring to assess exposures; these original studies therefore may have missed important intraurban gradients in exposure (Jerrett et al., 2005) and mischaracterize the true concentration‐response relationship. Lastly, it is important to note that “neighborhood” level characteristics of social disadvantage may not reflect individual level characteristics. For example, it is possible for a “low disadvantage” person to live in a “high disadvantage” neighborhood.

Additionally, there are some uncertainties inherent in using chemical transport models to assess ambient concentrations of PM_2.5_ and O_3_. Model predictions are provided at a 4‐km resolution, and hence, the model cannot resolve gradients or hotspots within the 4 × 4 km grid cell that might result at higher spatial resolutions. Regardless of the model performance of the absolute concentrations at a specific site, the model offers fairly robust predictions to changes in pollutant concentrations and captures broad urban‐rural spatial gradients (Yu et al., 2018). Additionally, we assumed that the average exposure estimates for the modeled periods (roughly 60 days per year) implemented in the study represents a consistent long‐term exposure for the study population. This assumes that sources and concentrations of exposure have remained fairly stable over time (retrospectively and into the future) and that the population is static. These underlying assumptions are common to quantitative HIA studies, as well as epidemiologic studies, but nonetheless may cause some uncertainty in the estimates of avoided impacts.

## 5 Conclusions

Overall, we found that reducing emissions from the two power plants in the southern Front Range region of Colorado would have modest health benefits for the region, and that these benefits would be realized primarily by residents living in ZCTAs with lower median incomes. Our results suggest that replacing these facilities with sources that do not increase emissions would have a positive environmental justice impact for Colorado residents. Our findings that the health benefits varied more by educational attainment and English proficiency than indicators of race or ethnicity highlight the need for local decision makers to carefully examine the demographics and socioeconomic status of potentially impacted groups and ensure that all appropriate indicators of social disadvantage are included in any policy evaluations. This is particularly important for states like Colorado which are less racially or ethnically diverse than other states or the nation as a whole.

## Conflict of Interest

The authors declare no conflicts of interest relevant to this study.

## Supporting information

Supporting Information S1
