# Supplementary material for: Health and Environmental Justice Implications of Retiring Two Coal‐Fired Power Plants in the Southern Front Range Region of Colorado
Source: Geohealth. 2019 Sep 26;3(9):266–83. doi: 10.1029/2019GH000206 (PMC7007175; doi:10.1029/2019GH000206)
Supplement: Supplementary file 1 — Supporting Information S1 [file GH2-3-266-s001.docx]

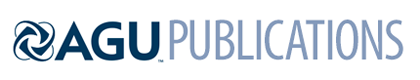


*GeoHeath*

Supporting Information for

Health and environmental justice implications of retiring two coal-fired power plants in the southern Front Range region of Colorado

**Sheena E. Martenies^1^, Ali Akherati^2^, Shantanu Jathar^2^, Sheryl Magzamen^3,4^**

^1^ Department of Environmental and Radiological Health Sciences, Colorado State University

^2^ Department of Mechanical Engineering, Colorado State University

^3^ Department of Epidemiology, Colorado School of Public Health

**Contents of this file**

Text S1 to S4

Figures S1 to S10

Tables S1 to S4

**Introduction**

The following sections include additional details on the health impact function inputs. The tables and figures also summarize the results of the alternative analysis (Health Benefits Scenario 2), which uses baseline emissions from the 2011 inventory and does not account for new controls installed at the Martin Drake facility in 2016.

Text S1. Health Outcome Incidence Rates

We calculated area-specific health outcome incidence rates for mortality and hospitalizations. Mortality data from the years 2010-2014 were obtained from the Colorado Department of Public Health and Environment. Each record contained information on year of death, primary cause of death identified on the death certificate as defined by the International Classification of Disease 10th Revision (ICD-10), the ZIP code of residence at death and age at death. For our analysis, we assumed ZIP codes and ZCTA were equivalent. Mortality counts were aggregated to the ZCTA level and used to calculate crude five-year average morality rates for the population 30 years of age or older for two sets of causes based on ICD-10 code: all-cause mortality (includes all ICD-10 codes) and non-accidental mortality (includes ICD-10 codes A00 – R99). The designation of “non-accidental” mortality was based on an environmental epidemiology study of the effects of ozone exposure on mortality (M. Bell, McDermott, Zeger, Samet, & Dominici, 2004). ZCTA populations were based on the 2014 American Community Survey (ACS) 5-Year Estimates (US Census Bureau, 2014).

Hospitalization data were obtained from the Colorado Hospital Association. Records from the years 2010-2014 were available, and each record contained information on year of the hospitalization, primary diagnosis defined by ICD-9 codes, the ZIP code of residence, and age at the time of the hospitalization. Again, we assumed ZIP codes and ZCTA were equivalent. These hospitalization data were aggregated to the ZCTA level and used to calculate crude 5-year average hospitalization rates for the population 65 years of age or older for two sets of causes based on ICD-9 codes: cardiovascular diseases (ICD-9 codes 390–459) and respiratory diseases (ICD-9 codes 460–519). Again, we used the ACS 5-Year estimates at the ZCTA level to calculate crude incidence rates for hospitalizations (US Census Bureau, 2014).

There are several other health outcomes that are causally related to PM_2.5_ and ozone exposures for which area-specific incidence data are not available. These outcomes include: emergency department visits for asthma among children (less than 18 years of age), asthma symptom days among children (i.e., days with cough, wheeze, or shortness of breath), missed days of school among school-aged children (6 to 18 years), minor restricted activity days (adults ages 18 and older), and work loss days (for adults ages 18-64). We took baseline incidence rates for these outcomes from the BenMAP User Manual and assigned them to each of the ZCTA in the study area (US Environmental Protection Agency [US EPA], 2015a).

Eq. 1 (main manuscript) requires the use of daily incidence rates to estimate the number of cases attributable to change in daily exposure concentrations. To estimate daily incidence rates, we assigned each ZCTA an annual rate (either the 5-year average annual rates calculated at the ZCTA level from the mortality and hospitalization data or the national-level rates from BenMAP) and then divide by 365 to obtain a daily rate.

Text S2. Concentration-Response Functions

CR functions were based on existing environmental epidemiology studies of the health effects of exposure to PM_2.5_ and O_3_. For this study, we relied on studies identified by US EPA (2015a). BenMAP used US EPA criteria for study design, validity, and generalizability when choosing studies from the large body of air pollution epidemiology (US EPA, 2009, 2012b, 2013, 2015a, 2015b). For premature mortality due to PM_2.5_ we used the CR estimate from the American Cancer Society study (Krewski et al., 2009); this CR function is the most widely cited study in the HIA literature. For all other impacts, we used pooled estimates from multiple studies based on methods reported in the most recent Regulatory Impact Analysis for the PM_2.5_ NAAQS (US EPA, 2012b). This pooling approach, which weighted each of the study-specific CR coefficients by the precision (i.e., the standard error) of the coefficient, generated an “average” coefficient for each pollutant-outcome pair. Pooling across several studies is useful when existing epidemiology studies may be small, conducted in areas outside of the HIA study area, or have limited generalizability (Hubbell et al., 2009). The studies used in this HIA along with the pooled concentration-response coefficients are listed in Table S1.

Text S3. Exposure Assessment

Exposures were assigned to each ZCTA using ordinary kriging in R. The exposure assessment methodology is illustrated in Figure S2. First, concentrations were predicted at known locations (4 km x 4 km) using the CMAQ model and then summarized to daily and monthly concentrations at each point. A subset of grid cells from the full CMAQ grid overlapping the study ZCTAs was selected and used in the exposure assessment (Figure S2). Next, concentrations were predicted for a more dense grid (1 km x 1 km) using the ordinary kriging prediction model. Finally, a population-weighted average of all the estimated concentrations that fall within a ZCTA is generated for each day and the entire month. This population weighted average is assigned to the entire ZCTA population.

For ZCTAs that fall along the edge of the CMAQ grid and are not completely covered by the grid, we assumed the population-weighted concentration estimated for the portion of the ZCTA that overlaps with the grid is applicable to the entire ZCTA. Our approach was reasonable given that most of the ZCTAs along the edge of the study grid had low population densities and pollutant concentrations.

Text S4. Exposed Populations

The number of exposed persons in each ZCTA was taken from the 2010-2014 ACS 5-year estimates (US Census Bureau, 2014). We assumed the entire population was exposed in each scenario. Exposure were based on the residential ZCTA of each person in the study area, which is consistent with most of the epidemiology studies used to generate the concentration-response functions used in this study. Although this approach may potentially bias the health impact estimates (Tchepel & Dias, 2011), individual-level data on commuting patterns and other time-activity data were not available for this study population.

For each pollutant-outcome pair, we limited the exposed population to those in the relevant age groups; for example, we assessed premature mortality only for the population in each ZCTA that is 30 years of age or older, and asthma symptom days are only assessed for children ages 7 to 14. This was done to match the population age groups used in the original studies; CR coefficients for one population age group may not be appropriate for other age groups. Stratification of the population was done using the age- and sex-specific data available from the ACS (US Census Bureau, 2014).


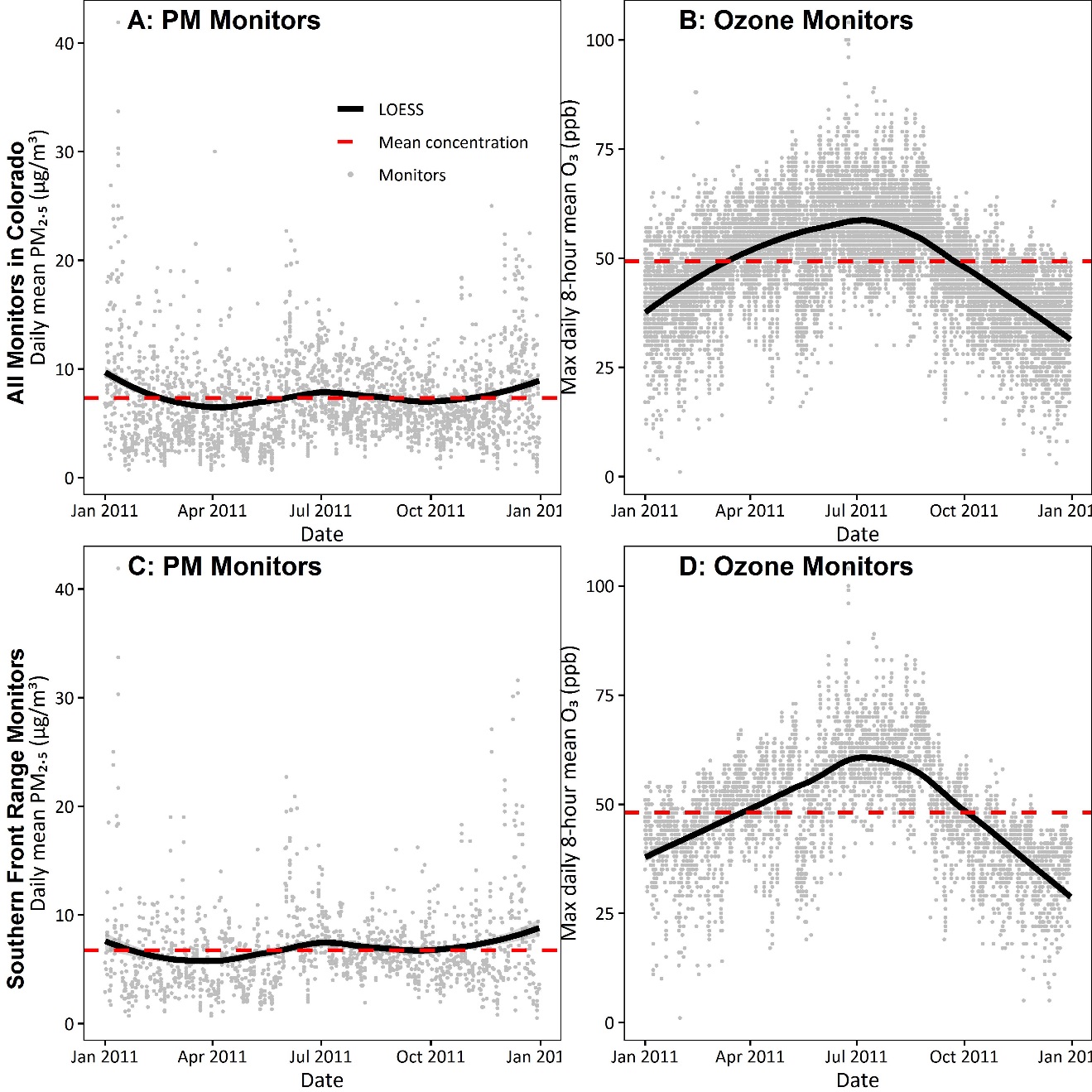
Figure S1. Time series plot of concentrations recorded in 2011 at all PM_2.5­_ (n = 19) and all O_3_ (n = 47) monitors in Colorado (A, B) and the subset of all PM_2.5­_ (n = 9) and O_3_ (n = 11) monitors located in the southern Front Range region of Colorado (C, D). The red dashed line shows the mean concentration across all monitors in 2011.


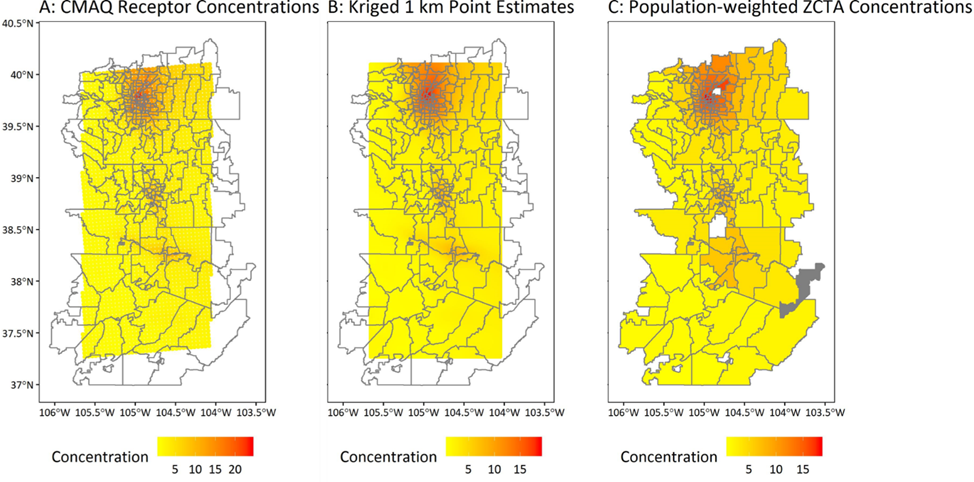


**Figure S2**. Example illustration of the exposure assessment methodology used in the health impact assessment


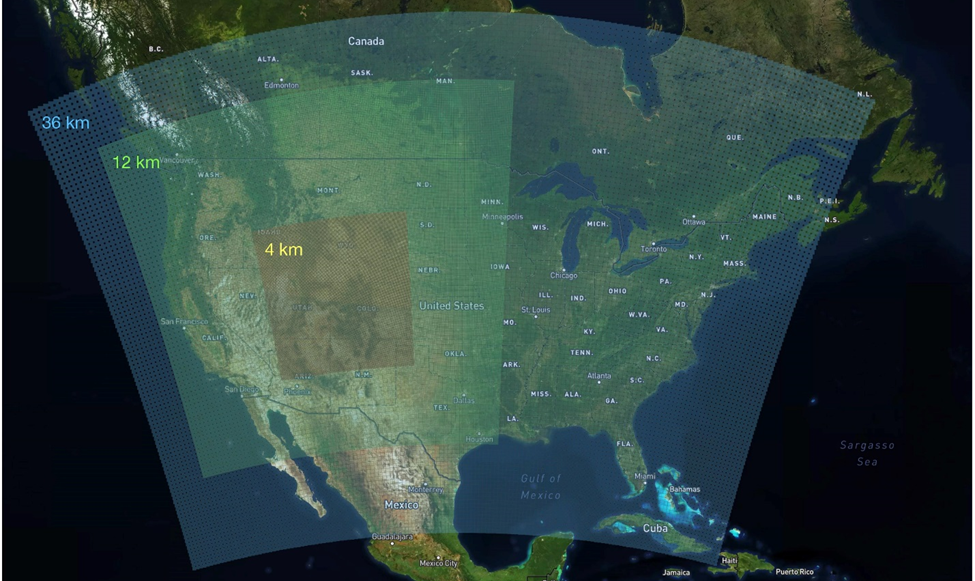


**Figure S3**. Full domain of the CMAQ model used to estimate the changes in ambient concentrations for each of the shutdown scenarios


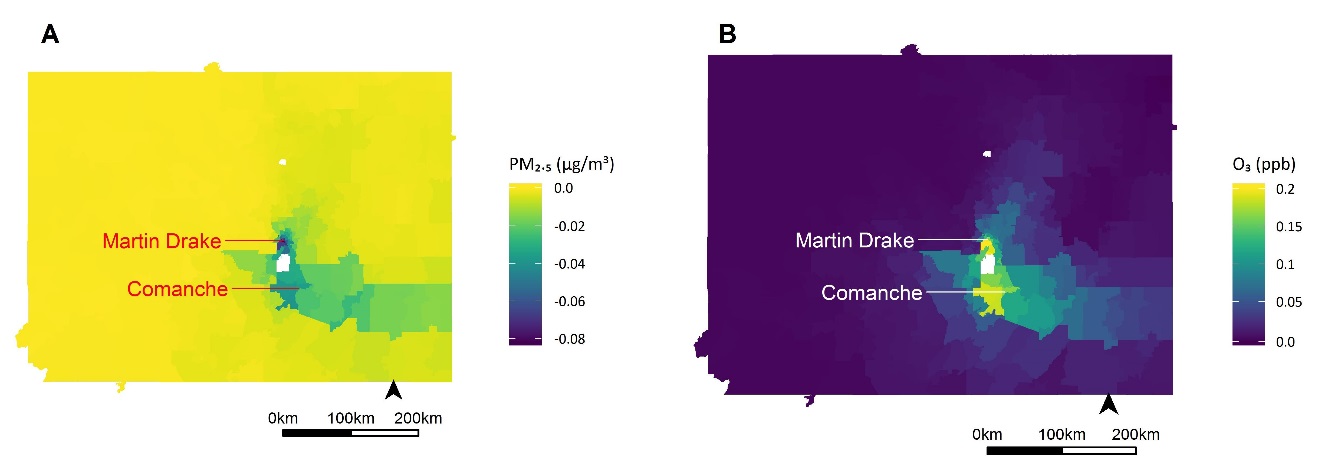


**Figure S4**. Changes in mean winter PM_2.5_ (A; µg/m^3^) and O_3_ (B; ppb) concentrations at the ZCTA level for Health Benefits Scenario 1.

**
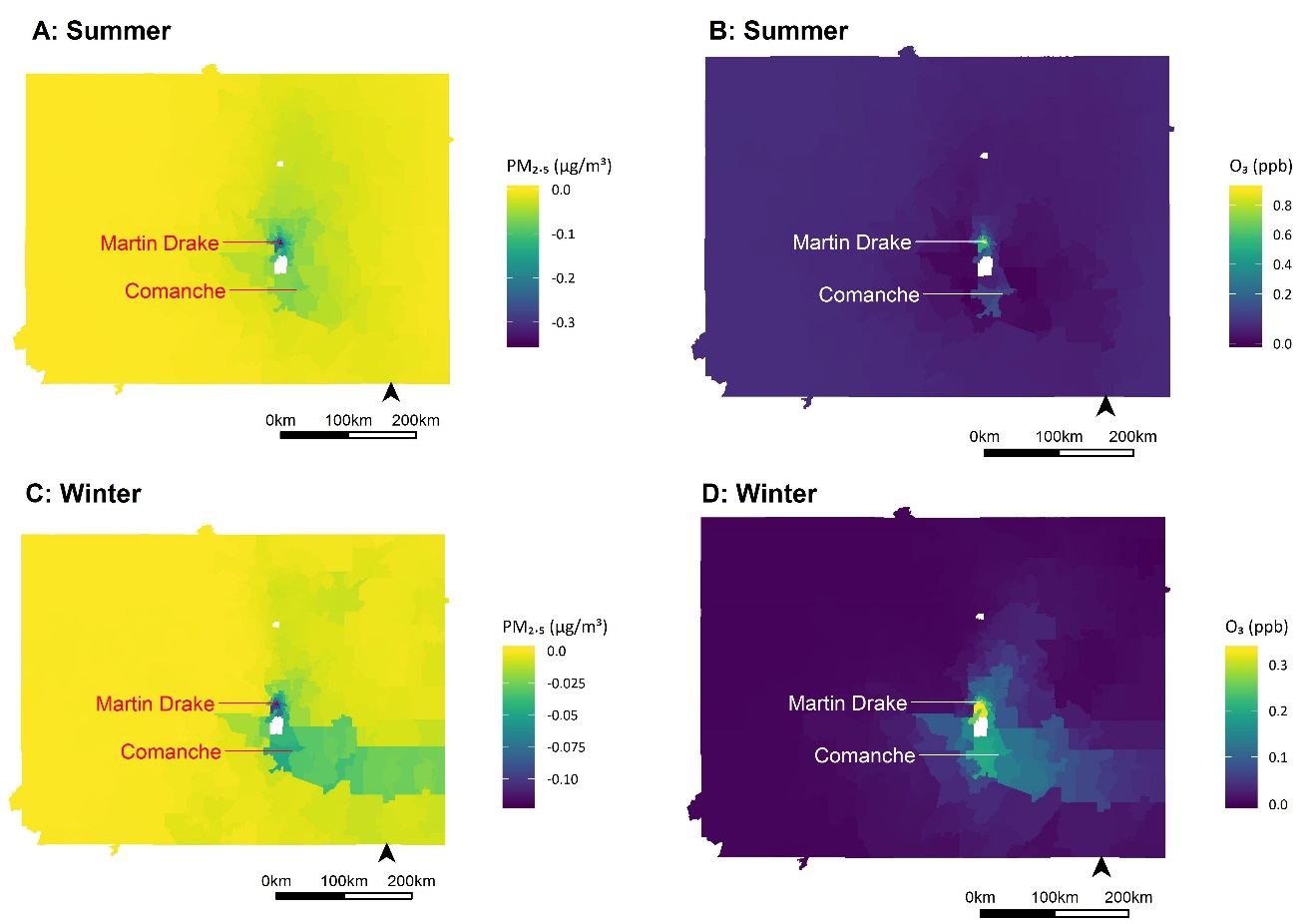
**

**Figure S5**. Changes in mean PM_2.5_ (µg/m^3^) and O_3_ (ppb) concentrations at the ZCTA level for each season modeled in Health Benefits Scenario 2


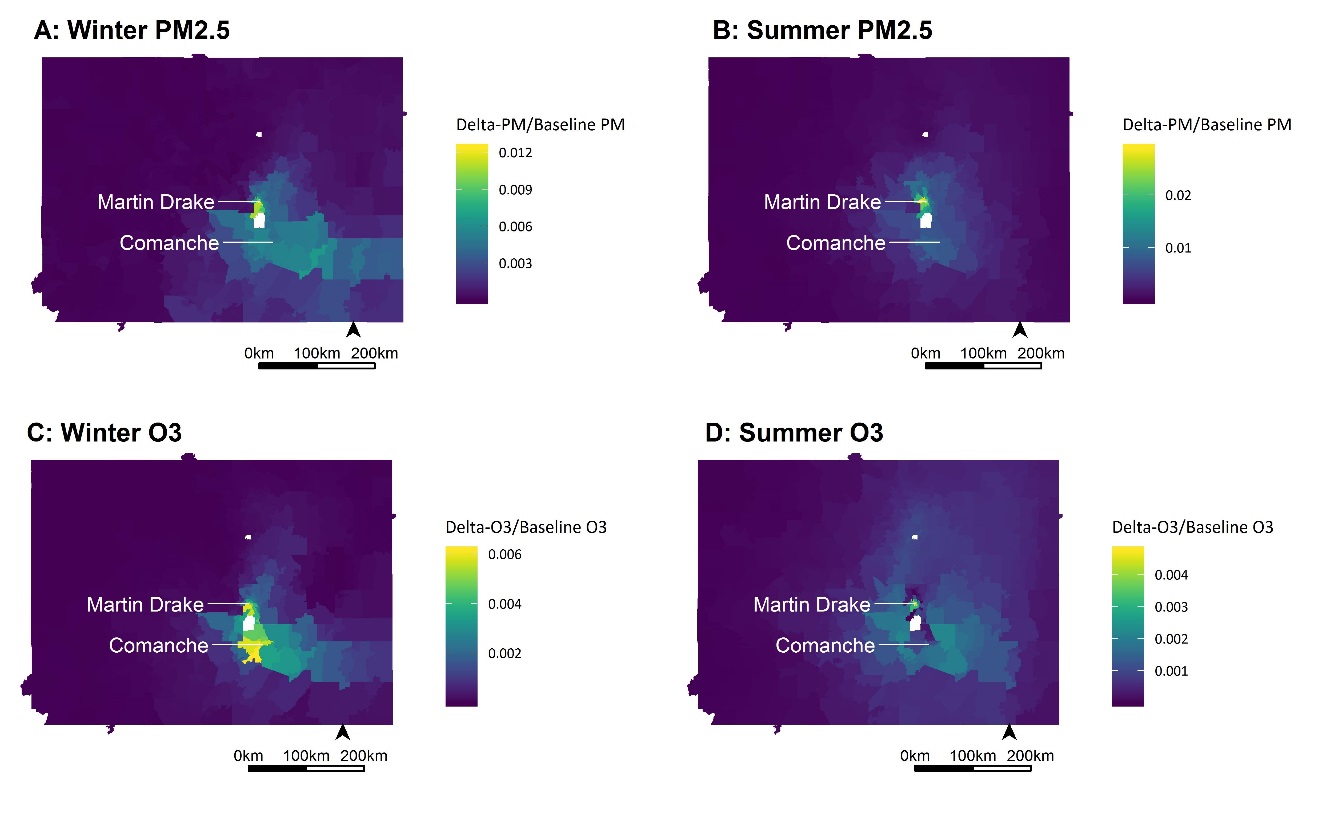


**Figure S6**. Maps showing the ratio of ΔPM_2.5­ ­_to baseline PM_2.55_ and ΔO_3_ to baseline O_3_ for the summer and winter seasons for Health Benefits Scenario 1.


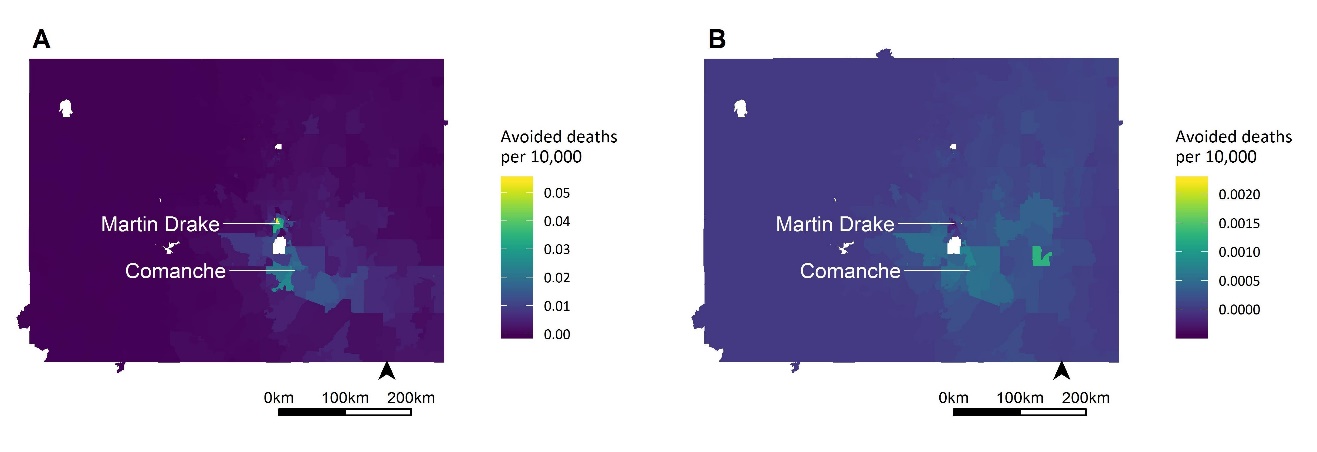


**Figure S7**. Maps of avoided mortality rates (per 10,000 persons per year) at the ZCTA level due to reductions in PM_2.5_ (A) and O_3_ (B) exposures for Health Benefits Scenario 1


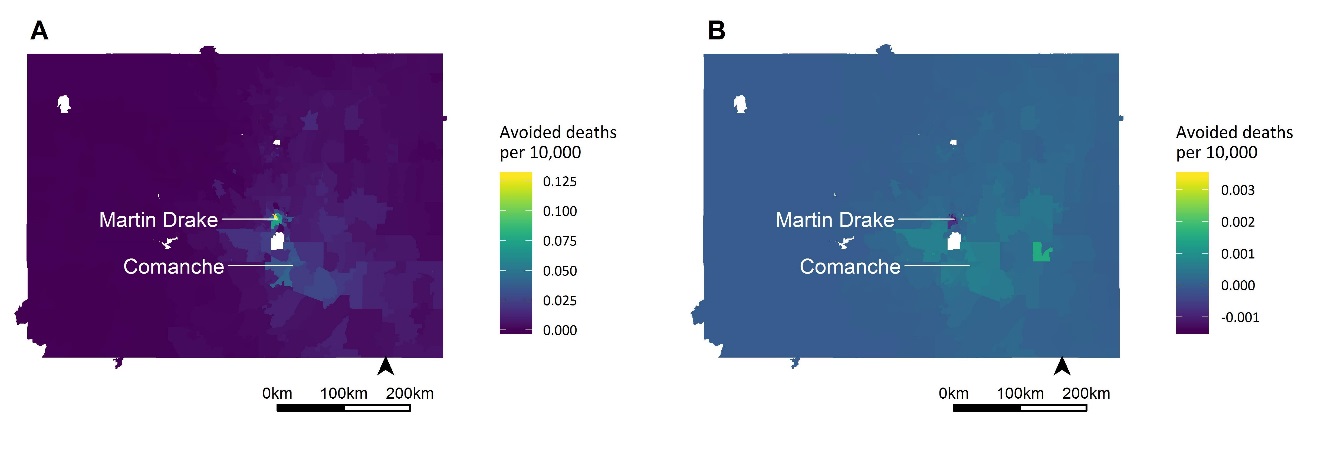


**Figure S8**. Maps of avoided mortality rates (per 10,000 persons) at the ZCTA level due to reductions in PM_2.5_ (A) and O_3_ (B) exposures for Health Benefits Scenario 2


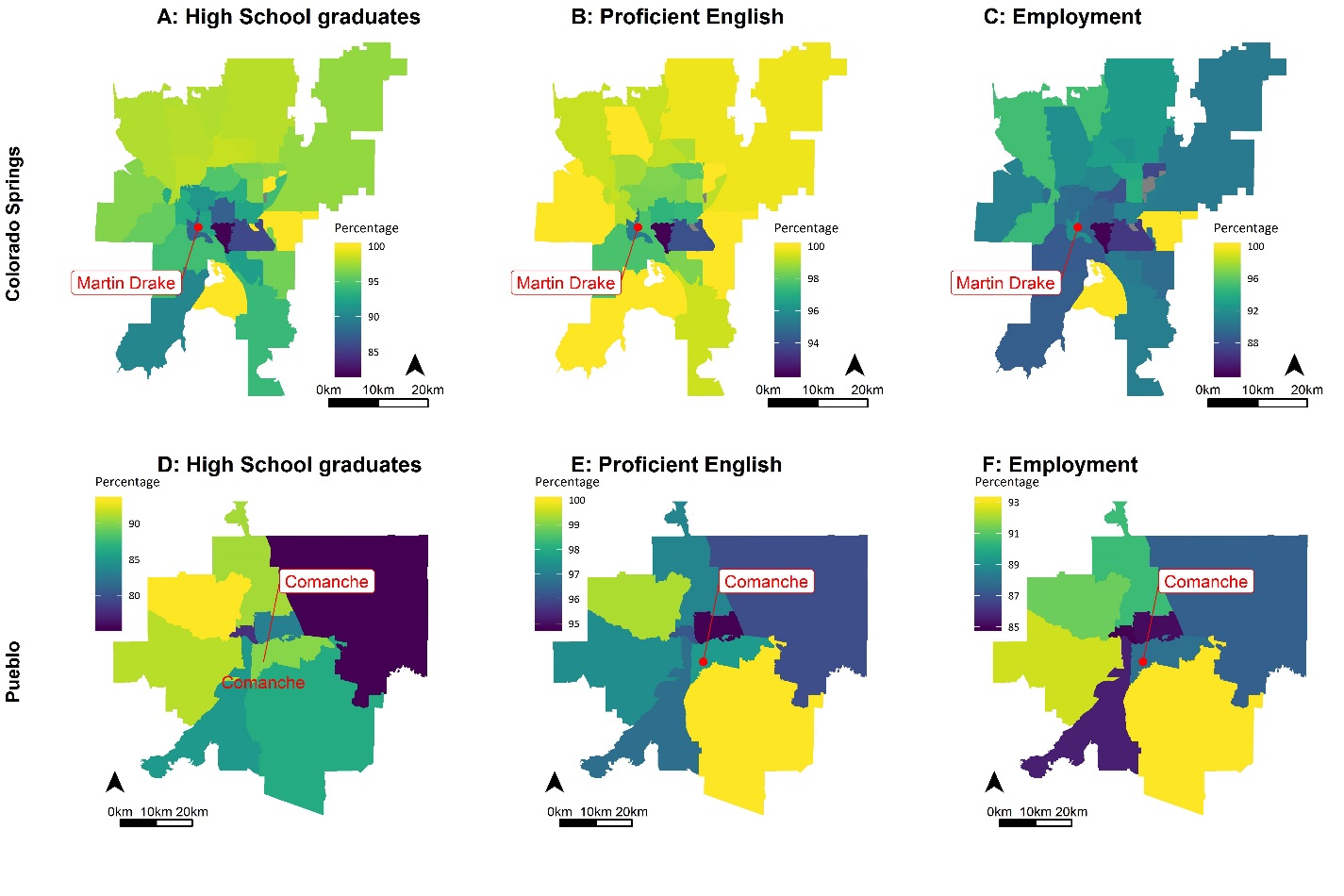


**Figure S9**. The percentage of ZCTA populations that are over the age of 25 without a high school diploma (or equivalent) (A, D), speak limited English (B, E) and are unemployed (C, F) at the ZCTA level for ZCTAs near the Comanche power plant in Pueblo, CO and near the Martin Drake power plant in Colorado Springs, CO.


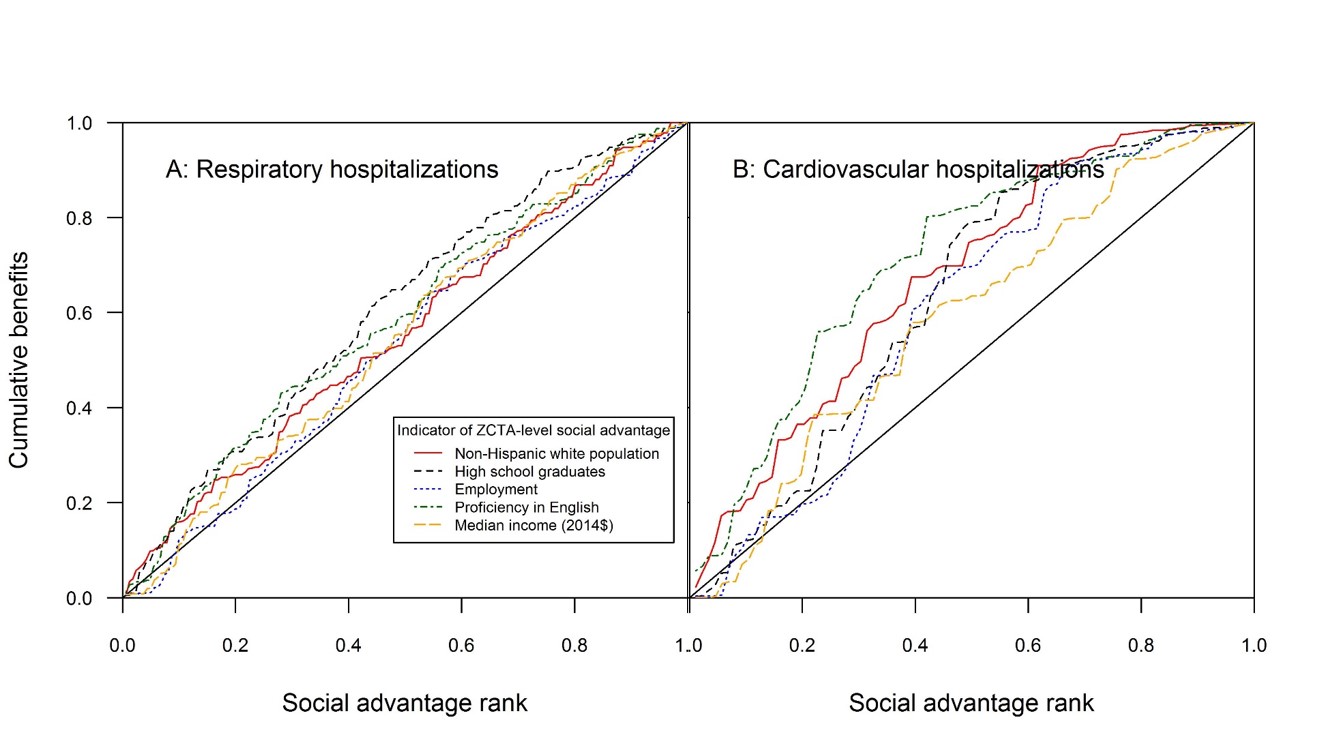


**Figure S10**. Concentration index curves for rates of avoided hospitalizations (per 10,000 persons) resulting from reduced PM_2.5_ or O_3_ exposures under Health Benefits Scenario 1 when ranking ZCTAs by indicators of social advantage

| Pollutant | Outcome Name | CR | SE of CR | | Source | Pooled CR | SE of Pooled CR | |
| --- | --- | --- | --- | --- | --- | --- | --- | --- |
| O_3_ | Asthma ED visit | 0.00306 | 0.00117 | | Glad et al., 2012 | 0.00288 | | 0.00098 |
|  |  | 0.00521 | 0.00091 | | Ito, Thurston, & Silverman, 2007 |  | |  |
|  |  | 0.00770 | 0.00284 | | Mar & Koenig, 2009 |  | |  |
|  |  | 0.01044 | 0.00436 | | Mar & Koenig, 2009 |  | |  |
|  |  | 0.00087 | 0.00053 | | Peel et al., 2005 |  | |  |
|  |  | 0.00111 | 0.00028 | | Sarnat et al., 2013 |  | |  |
|  |  | 0.00300 | 0.00100 | | Wilson, Wake, Kelly, & Salloway, 2005 |  | |  |
|  |  | -0.00100 | 0.00200 | | Wilson et al., 2005 |  | |  |
|  | Respiratory hospitalization | 0.00064 | 0.00040 | | Katsouyanni et al., 2009 | 0.00194 | | 0.00110 |
|  |  | 0.00178 | 0.00094 | | Schwartz, 1995 |  | |  |
|  |  | 0.00493 | 0.00177 | | Schwartz, 1995 |  | |  |
|  | Asthma symptom day | 0.00929 | 0.00387 | | Mortimer, Neas, Dockery, Redline, & Tager, 2002 | 0.00357 | | 0.00229 |
|  |  | 0.00097 | 0.00299 | | O’Connor et al., 2008 |  | |  |
|  |  | 0.00222 | 0.00282 | | Schildcrout et al., 2006 |  | |  |
|  | Minor restricted activity day | 0.00260 | 0.00078 | | B. D. Ostro & Rothschild, 1989 | 0.00260 | | 0.00078 |
|  | School loss day | 0.00158 | 0.00499 | | Lei Chen, 2000 | 0.00511 | | 0.00339 |
|  |  | 0.00815 | 0.00463 | | Gilliland et al., 2001 |  | |  |
|  | Non-accidental mortality | 0.00052 | 0.00013 | | Bell et al., 2004 | 0.00095 | | 0.00018 |
|  |  | 0.00039 | 0.00013 | | Bell et al., 2004 |  | |  |
|  |  | 0.00079 | 0.00013 | | Ito, De Leon, & Lippmann, 2005 |  | |  |
|  |  | 0.00172 | 0.00035 | | Ito et al., 2005 |  | |  |
|  |  | 0.00140 | 0.00027 | | Moolgavkar, Luebeck, Hall, & Anderson, 1995 |  | |  |
|  |  | 0.00140 | 0.00038 | | Moolgavkar et al., 1995 |  | |  |
|  |  | 0.00094 | 0.00030 | | Kelsall, Samet, Zeger, & Xu, 1997 |  | |  |
| PM_2.5_ | Asthma ED visit | 0.00516 | | 0.00324 | Glad et al., 2012 | 0.00473 | | 0.00148 |
|  |  | 0.00560 | | 0.00210 | Mar, Koenig, & Primomo, 2010 |  | |  |
|  |  | 0.00296 | | 0.00271 | Slaughter et al., 2005 |  | |  |
|  | CVD hospitalization | 0.00080 | | 0.00011 | Bell et al., 2008 | 0.00111 | | 0.00021 |
|  |  | 0.00140 | | 0.00034 | Moolgavkar, 2000 |  | |  |
|  |  | 0.00158 | | 0.00034 | Moolgavkar, 2003 |  | |  |
|  |  | 0.00071 | | 0.00013 | Peng, Chang, Bell et al., 2008 |  | |  |
|  |  | 0.00064 | | 0.00026 | Peng et al., 2009 |  | |  |
|  |  | 0.00187 | | 0.00028 | Zanobetti, Franklin, & Schwartz, 2008 |  | |  |
|  | Respiratory hospitalization | 0.00070 | | 0.00004 | Kloog, Coull, Zanobetti, Koutrakis, & Schwartz, 2012 | 0.00130 | | 0.00067 |
|  |  | 0.00205 | | 0.00044 | Zanobetti et al., 2008 |  | |  |
|  | Asthma symptom day | 0.01906 | | 0.00983 | Mar, Larson, Stier, Claiborn, & Koenig, 2004 | 0.08363 | | 0.77244 |
|  |  | 0.00099 | | 0.00075 | Ostro et al., 2001 |  | |  |
|  |  | 0.01222 | | 0.01385 | Mar et al., 2004 |  | |  |
|  |  | 0.00257 | | 0.00134 | Ostro et al., 2001 |  | |  |
|  |  | 0.00194 | | 0.00080 | Ostro et al., 2001 |  | |  |
|  | Minor restricted activity day | 0.00741 | | 0.00070 | Ostro & Rothschild, 1989 | 0.00741 | | 0.00070 |
|  | Work Loss Day | 0.00460 | | 0.00036 | Ostro, 1987 | 0.00460 | | 0.00036 |
|  | All-cause mortality | 0.00583 | | 0.00096 | Krewski et al., 2009 | 0.00583 | | 0.00096 |

Table S1. Summary of pollutants and health outcomes included in the HIA, studies from which concentration-response coefficients were taken, and pooled concentration-response coefficients used in the health impact functions.

| Pollutant | Season | Metric | Mean (SD) | Min | Median | Max |
| --- | --- | --- | --- | --- | --- | --- |
| PM_2.5_ | Winter | Monthly mean | -0.007 (0.014) | -0.120 | -0.003 | 0 |
|  | Winter | Daily mean | -0.007 (0.022) | -0.387 | 0.000 | 0.02 |
|  | Summer | Monthly mean | -0.022 (0.035) | -0.348 | -0.013 | 0 |
|  | Summer | Daily mean | -0.022 (0.05) | -0.952 | -0.004 | 0.003 |
|  |  |  |  |  |  |  |
| O_3_ | Winter | Monthly mean | 0.025 (0.053) | -0.003 | 0.004 | 0.332 |
|  | Winter | Daily mean | 0.026 (0.094) | -0.136 | 0.000 | 1.765 |
|  | Winter | Daily 1 hour max | -0.015 (0.056) | -0.717 | 0.000 | 0.372 |
|  | Winter | Daily 8 hour max | -0.003 (0.030) | -0.405 | 0.000 | 0.476 |
|  | Summer | Monthly mean | -0.025 (0.081) | -0.133 | -0.035 | 0.905 |
|  | Summer | Daily mean | -0.023 (0.132) | -0.820 | -0.006 | 2.415 |
|  | Summer | Daily 1 hour max | -0.244 (0.437) | -5.223 | -0.069 | 0.412 |
|  | Summer | Daily 8 hour max | -0.117 (0.198) | -2.494 | -0.039 | 0.367 |
| Abbreviations: PM_2.5_: particulate matter with an aerodynamic diameter less than 2.5 μm; O_3_: ozone; SD: standard deviation | | | | | | |

Table S2. Summary statistics for the change in population-weighted exposures to PM_2.5_ (µg/m^3^) and ozone (ppb) across all ZCTAs in the study area for Health Benefits Scenario 2.

|  |  | All ZCTAs | SFR | Colorado Springs | Pueblo |
| --- | --- | --- | --- | --- | --- |
|  | Outcome | Benefits per year | | | |
| PM_2.5_ | AC mortality | 5 (3, 7) | 3 (2, 4) | 2 (1, 3) | 0 (0, 1) |
|  | CVD hospitalization | 0 (0, 3) | 0 (0, 1) | 0 (0, 1) | 0 (0, 0) |
|  | RES hospitalization | 0 (0, 3) | 0 (0, 1) | 0 (0, 1) | 0 (0, 0) |
|  | ED visit for asthma | 0 (0, 0) | 0 (0, 0) | 0 (0, 0) | 0 (0, 0) |
|  | AST symptom day | 4 (-48, 130) | 4 (-25, 66) | 3 (-20, 53) | 0 (-3, 8) |
|  | MRAD | 72 (2, 540) | 50 (5, 250) | 41 (5, 200) | 6 (0, 30) |
|  | Work loss day | 12 (0,89) | 8 (1, 41) | 7 (1, 33) | 1 (0, 5) |
| O_3_ | NA mortality | 0 (0,1) | 0 (0, 0) | 0 (0, 0) | 0 (0, 0) |
|  | RES hospitalization | 0 (-17, 32) | -1 (-14, 8) | -1 (-9, 4) | 0 (-4, 3) |
|  | ED visit for asthma | 0 (0,0) | 0 (0, 0) | 0 (0, 0) | 0 (0, 0) |
|  | AST symptom day | 12 (-370, 1000) | 7 (-140, 330) | 4 (-87, 210) | 1 (-31, 65) |
|  | MRAD | 76 (-30, 840) | 31 (-27, 207) | 20 (-17, 130) | 7 (-7, 39) |
|  | School absence day | 80 (-92, 1300) | 37 (-58, 353) | 24 (-34, 220) | 8 (-16, 68) |
|  |  |  |  |  |  |
|  |  | Accrued benefits through 2035 | | | |
| PM_2.5_ | AC mortality | 95 (57, 133) | 57 (38, 76) | 38 (19, 57) | 0 (0, 19) |
|  | CVD hospitalization | 0 (0, 57) | 0 (0, 19) | 0 (0, 19) | 0 (0, 0) |
|  | RES hospitalization | 0 (0, 57) | 0 (0, 19) | 0 (0, 19) | 0 (0, 0) |
|  | ED visit for asthma | 0 (0, 0) | 0 (0, 0) | 0 (0, 0) | 0 (0, 0) |
|  | AST symptom day | 76 (-910, 2500) | 76 (-480, 1300) | 57 (-380, 1000) | 0 (-57, 150) |
|  | MRAD | 1400 (38, 10,000) | 950 (95, 4800) | 780 (95, 3800) | 110 (0, 570) |
|  | Work loss day | 230 (0, 1700) | 150 (19, 780) | 130 (19, 630) | 19 (0, 95) |
| O_3_ | NA mortality | 0 (0, 19) | 0 (0, 0) | 0 (0, 0) | 0 (0, 0) |
|  | RES hospitalization | 0 (-320, 610) | -19 (-270, 150) | -19 (-170, 76) | 0 (-76, 57) |
|  | ED visit for asthma | 0 (0, 0) | 0 (0, 0) | 0 (0, 0) | 0 (0, 0) |
|  | AST symptom day | 230 (-7000, 20,000) | 130 (-2700, 6200) | 76 (-1700, 3900) | 19 (-590, 1233) |
|  | MRAD | 1400 (-570, 16,000) | 590 (-510, 3900) | 380 (-320, 2400) | 130 (-130, 740) |
|  | School absence day | 1500 (-1700, 25,000) | 700 (-1100, 6700) | 460 (-650, 4200) | 150 (-300, 1300) |
| Abbreviations: AC: all-cause; AST: asthma; ED: emergency department; MRAD: minor-restricted activity day; NA: non-accidental; RES: respiratory; SFR: Southern Front Range | | | | | |

Table S3. Summary of annual and accrued health benefits (through 2035) as the median number of avoided premature deaths and cases of morbidity (2.5^th^ to 97.5^th^ percentiles) across the study area and for each subsection of the study area for Health Benefits Scenario 2.

|  |  | All ZCTAs | SFR | Colorado Springs | Pueblo |
| --- | --- | --- | --- | --- | --- |
|  | Outcome | Monetized benefits per year | | | |
| PM_2.5_ | AC mortality | 45,000 (27,000, 69,000) | 28,000 (16,000, 44,000) | 22,000 (12,000, 34,000) | 4,900 (3,000, 6,900) |
|  | CVD hospitalization | 17 (1, 126) | 12 (1, 61) | 9 (1, 46) | 2 (0, 11) |
|  | RES hospitalization | 11 (-1, 110) | 8 (-1, 53) | 6 (-1, 39) | 2 (0, 11) |
|  | ED visit for asthma | 0 (0, 0) | 0 (0, 0) | 0 (0, 0) | 0 (0, 0) |
|  | AST symptom day | 0 (-3, 8) | 0 (-2, 4) | 0 (-1, 3) | 0 (0, 0) |
|  | MRAD | 5 (0, 37) | 3 (0, 17) | 3 (0, 13) | 0 (0, 2) |
|  | Work loss day | 2 (0, 13) | 1 (0, 6) | 1 (0, 5) | 0 (0, 1) |
| O_3_ | NA mortality | 500 (-2,500, 8,000) | 120 (-2,100, 2,500) | -22 (-1,400, 1,300) | 87 (-540, 760) |
|  | RES hospitalization | 13 (-620, 1,200) | -27 (-520, 280) | -31 (-330, 140) | 1 (-150, 97) |
|  | ED visit for asthma | 0 (0, 0) | 0 (0, 0) | 0 (0, 0) | 0 (0, 0) |
|  | AST symptom day | 1 (-22, 62) | 0 (-8, 20) | 0 (-5, 12) | 0 (-2, 4) |
|  | MRAD | 5 (-2, 57) | 2 (-2, 14) | 1 (-1, 9) | 0 (0, 3) |
|  | School absence day | 8 (-9, 129) | 4 (-6, 35) | 2 (-3, 22) | 1 (-2, 7) |
|  |  |  |  |  |  |
|  |  | Accrued monetized benefits through 2035 | | | |
| PM_2.5_ | AC mortality | 700,000 (410,000, 1,100,000) | 430,000 (250,000, 670,000) | 330,000 (190,000, 520,000) | 74,000 (47,000, 110,000) |
|  | CVD hospitalization | 260 (8, 1,900) | 180 (19, 940) | 140 (19, 700) | 33 (1, 180) |
|  | RES hospitalization | 170 (-23, 1,700) | 120 (-12, 810) | 94 (-9, 590) | 24 (-2, 170) |
|  | ED visit for asthma | 0 (0, 1) | 0 (0, 1) | 0 (0, 0) | 0 (0, 0) |
|  | AST symptom day | 4 (-44, 120) | 3 (-23, 61) | 3 (-19, 49) | 0 (-3, 7) |
|  | MRAD | 75 (2, 560) | 52 (5, 260) | 43 (5, 210) | 6 (0, 31) |
|  | Work loss day | 27 (1, 210) | 19 (2, 95) | 16 (2, 75) | 2 (0, 11) |
| O_3_ | NA mortality | 7,600 (-38,000, 120,000) | 1,800 (-32,000, 38,000) | -340 (-22,000, 19,000) | 1,300 (-8,300, 12,000) |
|  | RES hospitalization | 210 (-9,500, 18,000) | -410 (-7,900, 4,300) | -480 (-5,100, 2,100) | 19 (-2,400, 1,500) |
|  | ED visit for asthma | 0 (0, 3) | 0 (0, 1) | 0 (0, 0) | 0 (0, 0) |
|  | AST symptom day | 11 (-340, 950) | 6 (-130, 300) | 4 (-80, 190) | 1 (-29, 60) |
|  | MRAD | 79 (-31, 870) | 33 (-28, 220) | 21 (-17, 130) | 7 (-8, 41) |
|  | School absence day | 120 (-140, 2,000) | 55 (-86, 530) | 36 (-51, 330) | 12 (-24, 100) |
|  |  |  |  |  |  |
|  | Total | 700,000 (360,000, 1,200,000) | 440,000 (210,000, 710,000) | 330,000 (160,000, 540,000) | 76,000 (37,000, 120,000) |
| Note: Monetized values are reported as 2011$ projected to a 2024 income level following methods reported by US EPA (US EPA, 2015a). Accrued benefits are discounted at a 3% rate. Values have been rounded to two significant digits.  Abbreviations: AC: all-cause; AST: asthma; ED: emergency department; MRAD: minor-restricted activity day; NA: non-accidental; RES: respiratory | | | | | |

**Table S4.** Summary of total monetized value of annual and accrued health benefits (through 2035) in $1,000’s (2.5^th^ to 97.5^th^ percentiles) across the study area and for each subsection of the study area for Health Benefits Scenario 2.
